# Combining lipidomics and machine learning to identify lipid biomarkers for nonsyndromic cleft lip with palate

**DOI:** 10.1172/jci.insight.186629

**Published:** 2025-05-08

**Authors:** Shanshan Jia, Weidong Xie, Chunqing Yang, Yizhang Dong, Wenting Luo, Hui Gu, Xiaowei Wei, Wei Ma, Dan Liu, Songying Cao, Yuzuo Bai, Wei Li, Zhengwei Yuan

**Affiliations:** 1 Key Laboratory of Health Ministry for Congenital Malformation, Shengjing Hospital of China Medical University, Shenyang, China.; 2 School of Computer Science and Engineering, Northeastern University, Shenyang, China.; 3 Department of Neurosurgery and; 4 Department of Pediatric Surgery, Shengjing Hospital of China Medical University, Shenyang, China.; 5 Key Laboratory of Intelligent Computing in Medical Image, Northeastern University, Shenyang, China.

**Keywords:** Development, Metabolism, Cholesterol, Embryonic development

## Abstract

Nonsyndromic cleft lip with palate (nsCLP) is a common birth defect disease. Current diagnostic methods comprise fetal ultrasound images, which are mainly limited by fetal position and technician skills. We aimed to identify reliable maternal serum lipid biomarkers to diagnose nsCLP. Eight-feature selection methods were used to assess the dysregulated lipids from untargeted lipidomics in a discovery cohort. The robust rank aggregation algorithm was applied on these selected lipids. The data were subsequently processed using 7 classification models to retrieve a panel of 35 candidate lipid biomarkers. Potential lipid biomarkers were evaluated using targeted lipidomics in a validation cohort. Seven classification models and multivariate analyses were constructed to identify the lipid biomarkers for nsCLP. The diagnostic model achieved high performance with 3 lipids in determining nsCLP. A panel of 3 lipid biomarkers showed great potential for nsCLP diagnosis. FA (20:4) and LPC (18:0) were also significantly downregulated in early serum samples from the nsCLP group in the additional validation cohort. We demonstrate the applicability and robustness of a machine-learning algorithm to analyze lipidomic data for efficient and reliable biomarker screening.

## Abstract 2

This study provides an approach for the early identification of foetal nsCLPs based on untargeted and targeted lipidomics combined with the machine learning algorithm.

## Graphical abstract

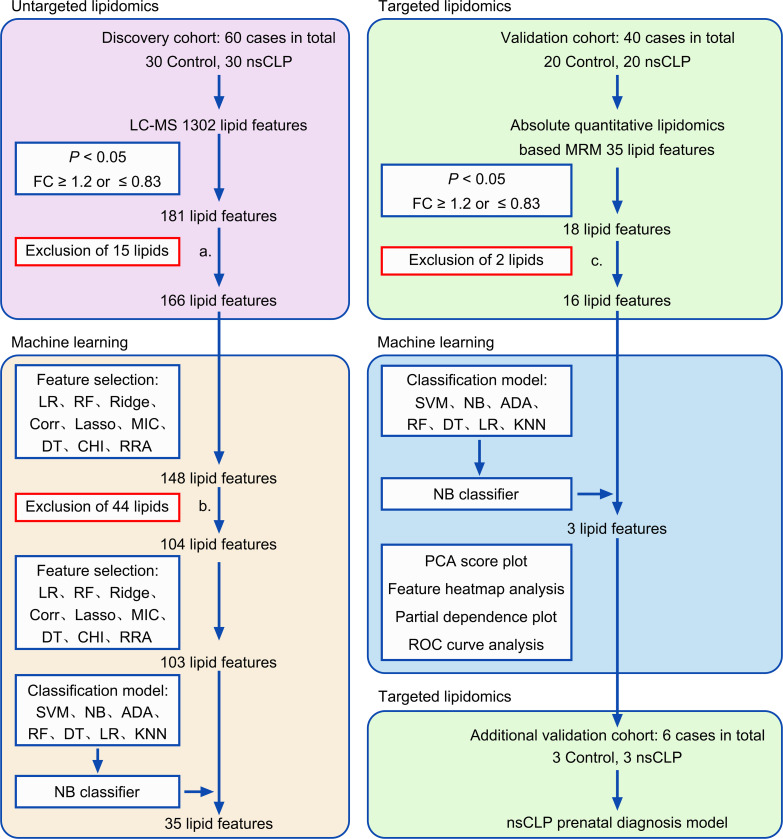

## Introduction

Nonsyndromic cleft lip with palate (nsCLP) is a common orofacial abnormality with a global incidence of 1 per 700 live births (1). Children with nsCLP are faced with hearing, eating, speech, and dental problems that require multiple surgeries, inducing a heavy financial burden and lifetime psychological problems for patients and families (2, 3). The exact etiology of nsCLP involves a combination of genetic and environmental risk factors (4). Metabolic abnormalities, including maternal diabetes and obesity, increase the risk of orofacial abnormality (5, 6). Current clinical strategies to diagnose nsCLP prenatally rely on ultrasound as early as 18–24 weeks of pregnancy (7). In China, the common gestational age for ultrasound is 22–26 weeks, depending on the consensus of experts and clinical practice. Ultrasound scan is performed in large prenatal diagnosis centers, and the success of examination is profoundly limited by maternal obesity, fetal position, equipment quality, and technician skill (2). More importantly, the palate is often not adequately visualized for acoustic shadowing, resulting in misdiagnoses of the cleft palate, and appropriate diagnoses are often not established until after birth (8).

Liquid biopsy-based tests have emerged as promising noninvasive diagnostic methods for congenital malformations. In clinical practice, maternal serum α-fetoprotein is used to screen for fetal neural tube defects. Such strategies have also been used for the prenatal diagnosis of fetal chromosomal aneuploidies, including detection of cell-free fetal DNA in maternal plasma. However, effective prenatal biomarkers for other congenital malformations, including nsCLPs, are still lacking. Prenatal diagnosis can give expecting parents time to prepare psychological and financial support for multidisciplinary surgical interventions, helping them avoid termination of the pregnancy. Human fetuses can heal from these interventions without visible scarring (9), and prenatal diagnosis combined with the development of fetal surgery and other therapeutic strategies could provide potential benefits for fetuses with nsCLP in the future. Therefore, a highly accurate, sensitive, and noninvasive serum testing strategy is urgently warranted to facilitate the prenatal diagnosis of nsCLP in clinical settings.

Compared with genomics, proteomics, and transcriptomics, lipidomics is still in its early stages. Owing to the high metabolite coverage of untargeted lipidomics and reliability of targeted lipidomics, the integration of both assays makes for a powerful disease-related lipid biomarker study (10). Recently, with the continuous development of efficient, high-throughput, and high-sensitivity analytical methods, lipidomics has been widely used to identify biomarkers in small volumes of plasma or serum for disease detection, prediction, and patient stratification (11, 12). Lipids contain several key components of the cell membrane structure, energy storage sources, and signaling molecules. Lipid homeostasis plays a critical role in supporting the physiological processes of cells and organisms. For instance, the dysregulation of lipid metabolism has been linked to fetal neurodegeneration and heart defects (13, 14). Long-chain polyunsaturated fatty acids (FA) and their metabolites are involved in the proper patterning of the dorsoventral axis of zebrafish (15). Our previous study found that 7 lipid biomarkers showed significant differences in patients following abortion and term birth after in vitro fertilization and embryo transfer (16). Palatal tissue lipidomics were analyzed in all-trans retinoic acid–treated cleft palate mice and normal mice, and unsaturated TGs were found to have a profound impact on palatogenesis (17). Embryonic development is strictly regulated by lipid metabolism, and lipid-related research is important for exploring biomarkers and the etiology of human nsCLP.

In the age of “big” data, machine learning (ML) is an important branch of artificial intelligence that can learn patterns from data; trained models can be used to predict outputs. The application of ML approaches may broaden and deepen our understanding of prenatal diagnosis, thus allowing us to improve delivery management and safety of mothers and newborns (18, 19). The combination of ML and lipidomics is an attractive and promising diagnostic approach. ML is utilized to recognize predictive patterns and actively used in lipidomics for data processing (20). Recent studies have illustrated that ensemble ML algorithms exhibit good performance in disease detection. An ensemble ML model of 9 individual classification models was built based on the maternal serum metabolomic profile to detect the presence of birth defects (21). The ensemble feature selection (ensemble-FS) strategy has proven to be efficient in selecting key features compared with typical feature selection methods (22). Li et al. designed an online tool based on ensemble-FS ranking to discover optimal proteomic features (23). However, no relevant studies have used the ensemble-FS strategy for lipidomic data analysis.

This study aimed to identify prenatal lipid biomarkers using a 2-step approach. First, a broad untargeted lipidomics approach was used in a discovery cohort, which has the potential to identify a wide range of lipid features. We employed the robust rank aggregation (RRA) approach to rank lipid features selected by 8-feature selection methods (24), followed by evaluating the RRA-ranked lipid features using 7 classification models. The second step involved a target lipidomics-based multiple-reaction monitoring (MRM) approach, in which the lipid features selected via the ML models in the first step are screened in the validation and additional validation cohorts. In this study, we evaluated the diagnostic accuracy of ML models based on maternal serum untargeted and targeted lipid signatures for the detection of prenatal lipid biomarkers of fetal nsCLP.

## Results

### Lipid profiling based on untargeted lipidomics.

**Figure 1:**
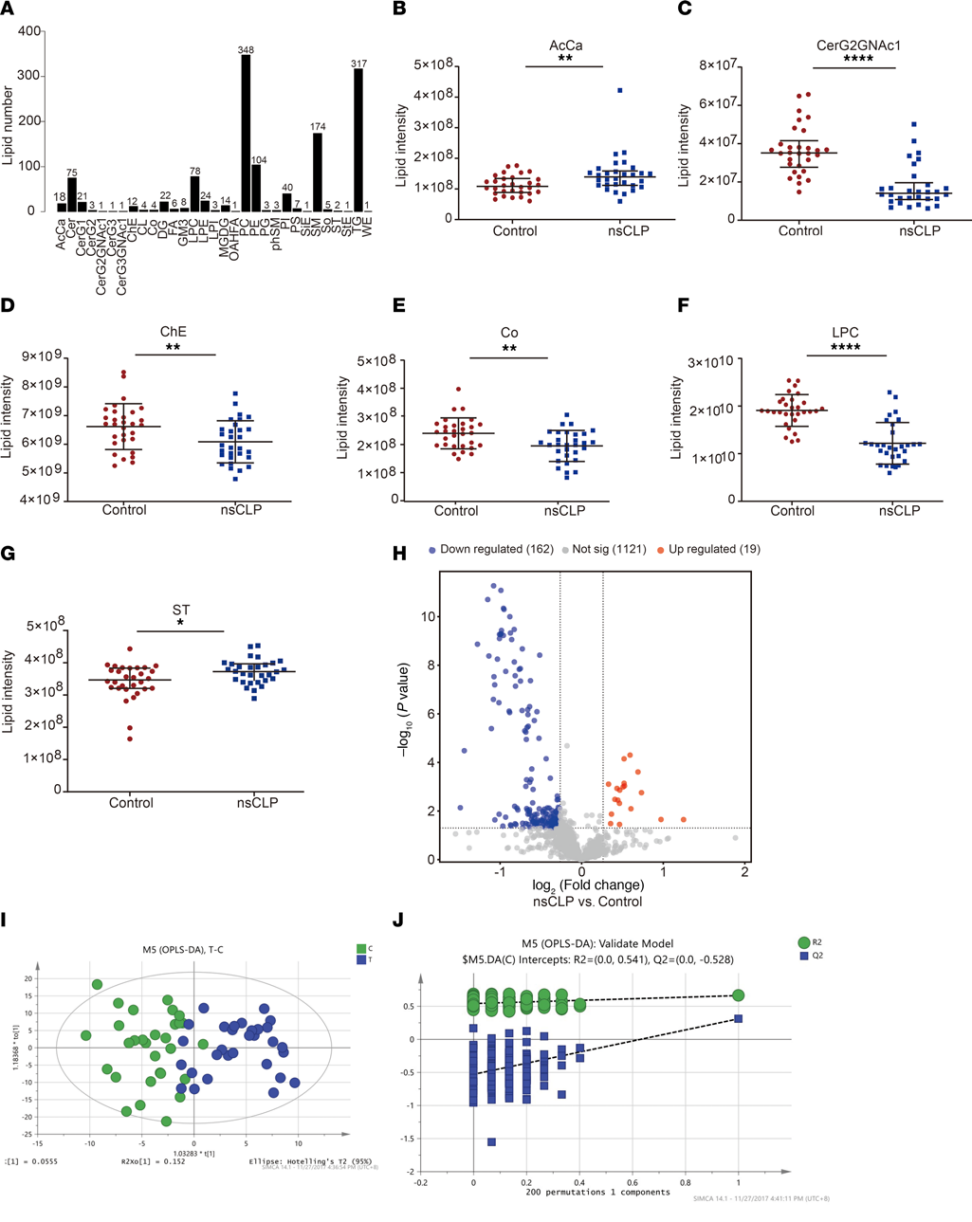
Serum lipid profiles identified by the untargeted lipidomics analysis. (**A**) A total of 1,302 lipid species were partitioned in 31 classes. (**B**–**G**) Lipid intensity of the 6 differentially dysregulated lipids classes (acyl carnitine [AcCa], CerG2GNAc1, cholesterol ester [ChE], coenzyme [Co], lysophosphatidylcholine [LPC], and sulfatide [ST]) in the control and nsCLP groups. Data are expressed as mean ± SD. *n* = 30, each group. *P < 0.05; **P < 0.01; ****P < 0.0001 by unpaired *t* test or median with interquartile ranges by Mann-Whitney test. (**H**) Volcano plot analysis of differentially expressed lipids in maternal serum from the control and nsCLP group (FC ≥ 1.2 or FC ≤ 0.83 and *P* < 0.05). (**I**) OPLS-DA score plot based on control (green dots) and nsCLP samples (blue dots) (*n* = 30, each group). (**J**) Statistical validation of the OPLS-DA model by permutation testing (200 iterations).

The distribution of the relative standard deviation (RSD) of the peak areas for all identified lipids in the quality control (QC) samples indicated that 98% in both positive and negative modes of the lipid features had an RSD of ≤ 30% (Supplemental Figure 1A; supplemental material available online with this article; https://doi.org/10.1172/jci.insight.186629DS1). Results showed satisfactory stability and repeatability of untargeted lipidomic analysis. A total of 31 lipid classes and 1,302 lipid species was detected in 60 serum samples collected at a gestational age of 22–26 weeks. Phosphatidylcholine (PC; *n* = 348), triglyceride (TG; *n* = 317), sphingomyelin (SM; *n* = 174), phosphatidylethanolamine (PE; *n* = 104), and lysophosphatidylcholine (LPC; *n* = 78) were the top 5 classes with the highest number of lipid species (Figure 1A). Among the untargeted lipidomic data, 6 lipid classes were significantly dysregulated in the sera of pregnant women carrying fetuses with nsCLP compared with pregnant individuals in the control group. Acyl carnitine and sulfatide were significantly upregulated, while CerG2GNAc1, cholesterol ester, coenzyme (Co), and LPC were significantly downregulated in the nsCLP group (Figure 1, B–G). In total, 1,302 lipids were detected, including 19 upregulated, 162 downregulated, and 1,121 nonsignificant lipids in the sera of pregnant women carrying fetuses with nsCLP compared with individuals in the control group (Figure 1H). The orthogonal partial least squares-discriminant analysis (OPLS-DA) model was established through 7-fold cross-validation to obtain a list of features for defining the variation in lipid profiles between the nsCLP and control groups (Figure 1I). Even though the permutation test of the OPLS-DA model showed no overfitting (R^2^ intercept was 0.541, and the Q^2^ intercept was –0.528; Figure 1J), the R^2^X, R^2^Y, and Q^2^ values were 0.395, 0.662, and 0.311, respectively, indicating that the stability and reliability of the OPLS-DA model were unsatisfactory. To further improve diagnostic accuracy, we subsequently used multiple ML approaches to establish optimal prenatal lipid biomarkers for fetal nsCLP.

### ML algorithm performance in untargeted lipidomics.

**Figure 2:**
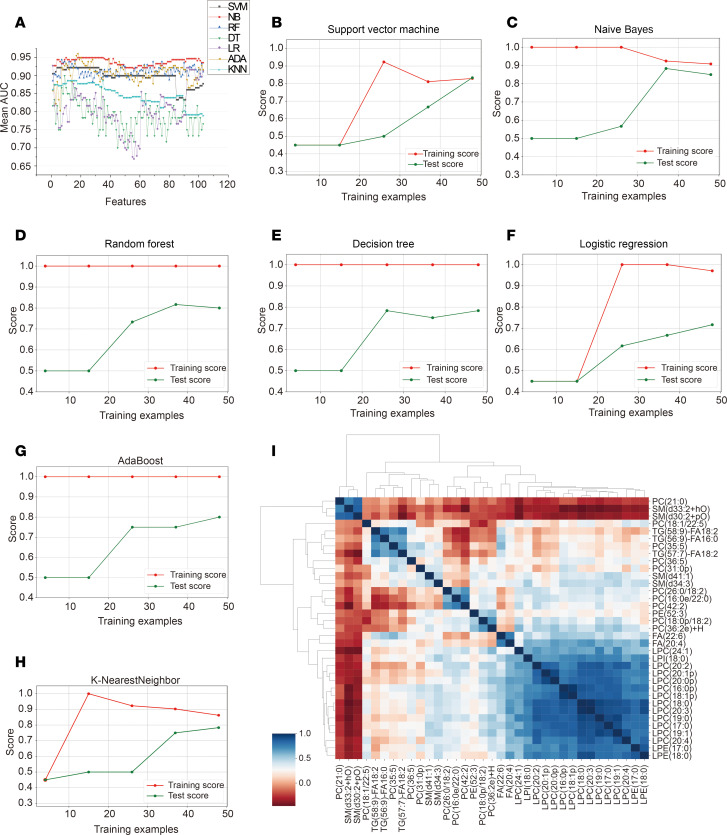
Classification model performance on the discovery cohort. (**A**) Analysis of the mean AUC changes in 103 lipid features under different classifiers. (**B**–**H**) Learning curve analysis of the 7 different classifiers, including SVM, NB, RF, DT, LR, ADA, and KNN. (**I**) Correlation clustering heatmap for these 35 lipid features identified via the NB classifier.

First, 8 feature evaluation methods were performed on the 181 differentially dysregulated lipid features (fold change [FC] ≥ 1.2 or ≤ 0.83, *P* < 0.05). Each algorithm was used to independently evaluate all lipid features, resulting in a feature-ranking list. After deleting lipid isomers and lipids that could not be validated using targeted lipidomics, 103 lipid features were included in the RRA ranking fusion approach. Subsequently, 103 lipid features were selected using the aforementioned ensemble-FS method. The details are provided in the Supplemental Data. Seven classification models were used to determine the diagnostic performance of the 103 lipid features. Figure 2A shows the mean AUC values of different lipid features based on different classification models. The highest AUC value among the individual models resulting from the naive Bayes (NB) classification model was 0.95, when the top 35 lipid features were included. Table 1 shows the diagnostic performances of the 35 lipid features for each classification model when applied to a discovery cohort. To further analyze the performance of the different classifiers, we plotted the training and testing curves for different classifiers using accuracy as the metric (Figure 2, B–H). These results also demonstrated the superiority of the NB classifier. The NB classification model did not pose any risk of overfitting, achieving high classification accuracy and a balanced distribution on the training and testing sets. Tree-based classification models, including random forest (RF), Decision tree (DT), and Adaboost (ADA), tended to achieve 100% classification accuracy on the training set; however, it was difficult to achieve a classification accuracy above 0.8 on the testing set, indicating poor performance and a risk of overfitting. However, the logistic regression (LR), support vector machine (SVM), and K-nearest neighbor (KNN) models did not achieve satisfactory accuracy values, even when all samples were included as inputs. The correlograms were represented by the Pearson correlation coefficient. Among all feature correlations, 75% (449 of 595) had a correlation coefficient of less than 0.5, reflecting relatively weak linear associations among the majority of features (Figure 2I).

### Multivariate analysis of candidate lipids biomarkers in the discovery cohort.

**Figure 3:**
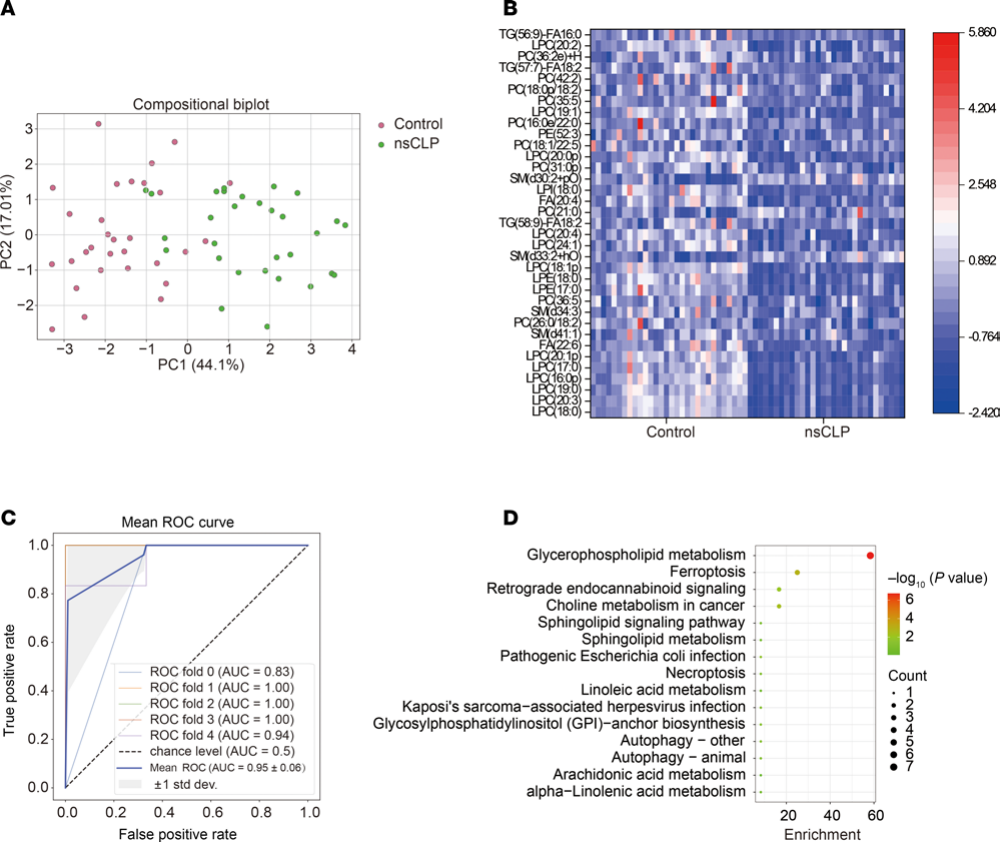
Differentially expressed lipids and their enriched metabolism pathways. (**A**) PCA score plot of 35 lipid features based on control (red dots) and nsCLP samples (green dots) (*n* = 30, each group). (**B**) Feature heatmap analysis of 35 lipid features in the control and nsCLP samples (*n* = 30, each group). (**C**) ROC curve analysis of the NB classifier of 35 lipid features. Data are presented as mean ± SEM. (**D**) Bubble plot shows 15 lipid metabolism pathways enriched by 35 lipids according to lipid hits and –log_10_ (*P* value).

The principal component analysis (PCA) model demonstrated a separation tendency of 35 lipid features between the control and nsCLP groups in the score plot (Figure 3A). A heatmap of the 35 selected features demonstrated that the expression levels in the nsCLP group were significantly different from those in the control group (Figure 3B). The average AUC values of NB classifier were repeatedly calculated 5 times for hierarchical cross-validation. In combination with the 35 RRA-selected lipid features, the NB classifier achieved optimal performance with an AUC value, sensitivity, and specificity of 0.95, 90%, and 87%, respectively, indicating high discrimination performance between the 2 groups (Figure 3C). Figure 3D showed 15 pathways enriched by 35 dysregulated lipid features, with bubble size and distance to origin varying with lipid counts and –log_10_ (*P* value), respectively. Five pathways with a *P* value of less than 0.05 — glycerophospholipid metabolism, ferroptosis, choline metabolism in cancer, retrograde endocannabinoid signaling, and pathogenic *E*. *coli* infection — were considered the most significantly enriched pathways. The detailed results of pathway enrichment are summarized in Supplemental Table 1.

### Performance of ML algorithms in relation to targeted lipidomics.

**Figure 4:**
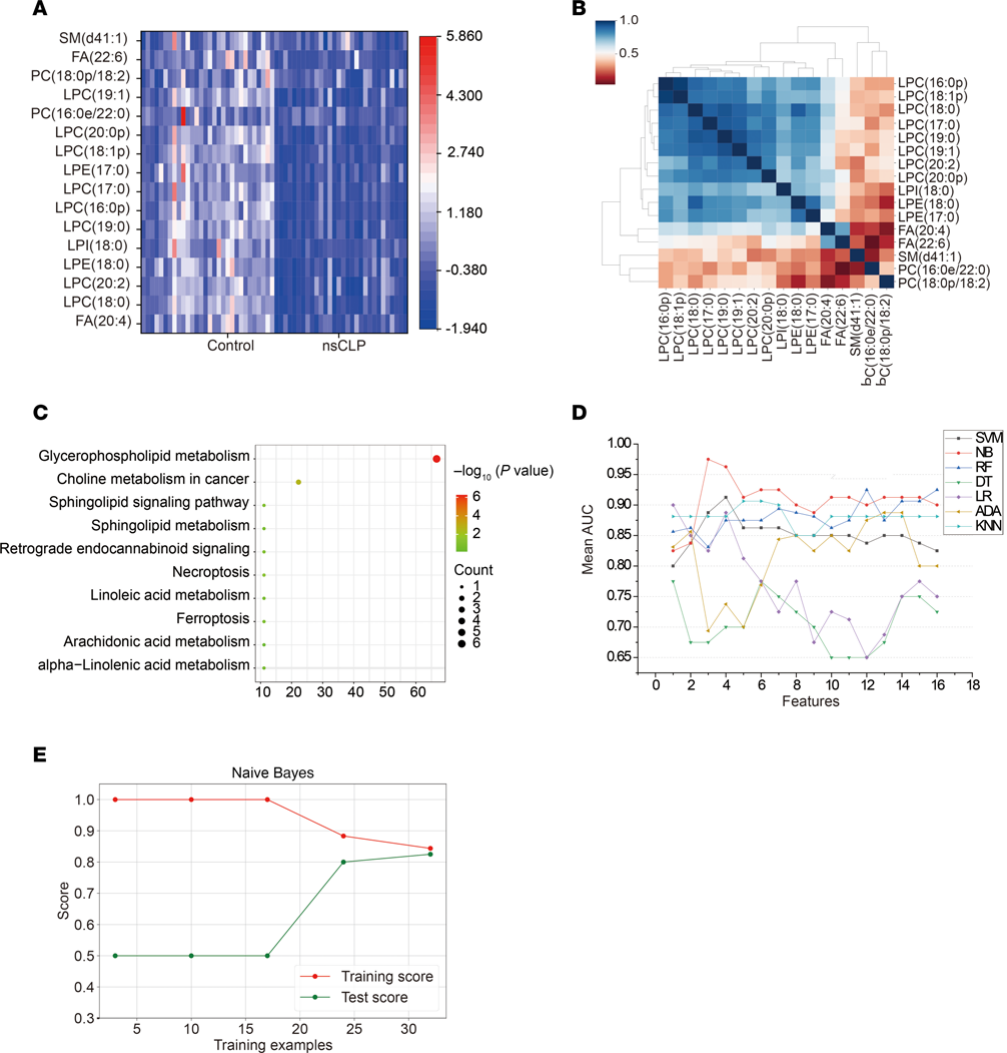
Functional analysis and classification model performance on targeted lipidomic data. (**A**) Feature heatmap analysis of 16 lipid features in the control and nsCLP samples (*n* = 20, each group). (**B**) Correlation clustering heatmap for these 16 lipid features identified by targeted lipidomics (*n* = 20, each group). (**C**) Bubble plot showing 10 lipid metabolism pathways enriched by 16 lipids according to lipid hits and –log_10_ (*P* value). (**D**) Analysis of the mean AUC changes in 16 lipid features under different classification models. (**E**) Learning curve analysis of the NB classifier.

The expression levels of 35 lipid features were tested using targeted lipidomic analysis in a validation study with an independent population, and 16 lipids were detected as differentially expressed features. Results revealed that the sum of the responses for these lipids with a QC RSD percentage of ≤ 30% reached 80%, demonstrating good stability for targeted lipidomics (Supplemental Figure 1B). A heatmap of the 16 selected features demonstrated that the expression levels in the nsCLP group were significantly different from those in the control group (Figure 4A). A correlogram showed that 8 LPC features were associated with metabolic disorders in the nsCLP group (Figure 4B). Two pathways (glycerophospholipid metabolism and choline metabolism in cancer) were significantly enriched in the bubble dot diagram (Figure 4C). The detailed results of pathway enrichment are summarized in Supplemental Table 2. Seven classification models were used to build the diagnostic performance of the 16 lipid features in the targeted analysis. Figure 4D shows the AUC values for lipid features based on different classification models. The highest AUC value among the individual models resulting from the NB classification model was 0.97, when the top 3 lipid features (FA [20:4], LPC [18:0], and PC [16:0e/22:0]) were included. Table 2 showed the diagnostic performance of each classification model when applied to the validation cohort. Consistent with our previous findings from the untargeted analysis, the NB classifier demonstrated a clear advantage among the 7 classification models (Figure 4E). The individual training curves of the other 6 classification models are shown in Supplemental Figure 2.

### Lipid features as prenatal biomarkers for nsCLP.

**Figure 5:**
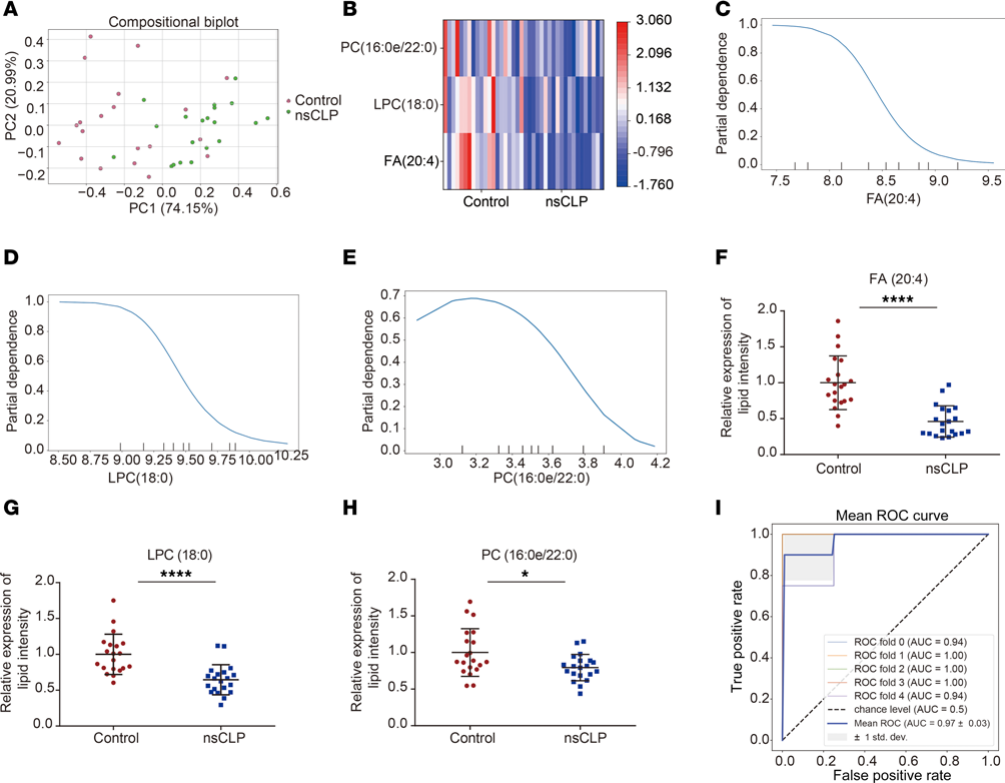
Three lipids biomarkers for nsCLP. (**A**) PCA score plot of 3 lipid features based on control (red dots) and nsCLP samples (green dots) (*n* = 20, each group). (**B**) Feature heatmap analysis of 3 lipid features (*n* = 20, each group). (**C**–**E**) PDP analysis for the 3 lipid features (FA [20:4], LPC [18:0], and PC [16:0e/22:0]). (**F**–**H**) Relative expression of the 3 lipid features in nsCLP compared with control group. Data are presented as mean ± SD. *n* = 20, each group. *P < 0.05; ****P < 0.0001, by unpaired *t* test. (**I**) ROC curve analysis of NB classifier of 3 lipid features. Data are presented as the mean ± SEM. *n* = 20, each group.

The PCA score plot of the 3 lipid features demonstrated significant separation between the control and nsCLP groups, indicating a dramatically dysregulated lipid profile (Figure 5A). Feature heatmap analysis showed that the 3 lipid features in the nsCLP group were significantly different from those in the control group (Figure 5B). Partial dependence plots (PDP) serve as a valuable tool for illustrating the impact of a specific feature on model predictions. To verify the validity of the results, we conducted a PDP analysis of the features selected using the NB classifier model. The 3 lipid features were obviously steep, indicating that the features had a substantial influence on prediction results (Figure 5, C–E). Therefore, these lipids contributed significantly to the model and validated the results. The expression levels of 3 lipid features (FA [20:4], LPC [18:0], and PC [16:0e/22:0]) were significantly downregulated in the nsCLP group (Figure 5, F–H). The mean AUC for the 3 lipid features was 0.97, with a sensitivity of 80% and specificity of 84% (Figure 5I). To explore the prenatal diagnosis efficiency of the 3 lipid biomarkers, we collected serum samples from women carrying fetuses with nsCLP in the early stages of pregnancy. The results showed that the expression levels of FA (20:4) and LPC (18:0) were also significantly downregulated in sera from the pregnant women carrying fetuses with nsCLP with gestational ages of 16–17 weeks compared with that in the control group (Supplemental Figure 3).

## Discussion

In this study, a combination of lipidomics and ML was used to screen serum lipid biomarkers for the diagnosis of fetal nsCLP. Three lipid features were ultimately identified as biomarkers, and a diagnostic model based on a panel of these 3 features was built and evaluated using a validation cohort. Our findings provide a noninvasive and efficient diagnostic method for fetal nsCLP as well as a study workflow that combines lipidomics and ML that can be used to efficiently and reliably screen for biomarkers. In the discovery cohort of this study, extensive lipid features were screened using untargeted lipidomic analysis. We employed 8-feature selection methods to rank all lipid features according to their relative contributions in discriminating the different groups. The RRA approach was subsequently used to generate a unified ranking list based on the selected features via the 8-feature selection methods. The 7 classification models provided good overall accuracy in terms of the selected lipid features ranked using the RRA approach. We further employed an NB-based classification model to screen the lipidomics for the following reasons. (a) The model is based on the assumption of the conditional independence of features to perform accurate classification with a small sample size; thus, it is suitable for our lipidomics (25). (b) The NB classifier is robust against noisy data: as the underlying algorithm is based on a probabilistic model, it is insensitive to noise or outliers in the input data (26). (c) In the present study, the NB classifier was ranked as the best among the 7 classification models, with the lowest number of lipid biomarkers in relation to AUC, sensitivity, and specificity. To validate the panel of lipid biomarkers and ultimately apply it in clinical settings, we performed targeted lipidomic analysis on 35 potential lipid biomarkers in a validation cohort and identified 16 differentially dysregulated lipid features. The 7 included classifications were used to analyze 16 lipid biomarkers, and the NB classifier was the optimal classifier in the validation cohort. Our results showed good diagnostic power in the validation cohort with the 3 lipid features.

In recent years, more and more studies have applied ML algorithms to medical applications, taking advantage of their remarkable sensitivity and promising potential for diagnosis. For example, Zhou et al. implemented an SVM-based ML algorithm to search a panel of 11 plasma lipids for malignant brain gliomas (27). ML approaches have also been used to identify and predict common birth defects, including congenital heart defects (28). Our previous study used an SVM classifier to establish a prediction model based on a combination of 3 complement proteins and α-fetoprotein, which could serve as optimal prenatal biomarkers for neural tube defects (29). This study employed a 2-stage ML approach. Initially, a classification model was developed to identify potential lipid biomarkers from untargeted lipidomics. Subsequently, a diagnostic model was constructed using targeted lipidomics from the validation cohort to develop a robust diagnostic framework for fetal nsCLP detection. To enhance the stability and effectiveness of biomarker selection and eliminate the randomness and bias caused by a single-feature selection method, we integrated 8-feature selection algorithms and used the RRA method to combine the results of different algorithms to obtain optimal feature ranking. To address various factors that may influence ranking lists in different feature selection methods, including individual errors and method preference differences, RRA transforms the ranking lists into rank values. This approach effectively mitigates the impact of noise and outliers, thereby improving overall robustness. In addition, we tested 7 classification models and identified the optimal model for diagnosing nsCLP.

The candidate lipid biomarkers belonged to 3 lipid classes (PC, LPC, and FA). PC represents a key molecular constituent of cellular membranes, pulmonary surfactants, circulating lipoproteins, and biliary secretions. The role of PC in emulsifying and decomposing lipids exerts a positive protective effect on the cardiovascular system (30). PC is a source of lipid messengers and can be catalyzed by secretory phospholipase A2 to produce LPC. These data further explain the results of the downregulated levels of both PC (16:0e/22:0) and LPC (18:0) in the present study. LPC represents a class of oxidized low-density lipoprotein components whose homeostasis is maintained through enzymatic regulation. This lipid class modulates cellular functions via apoptosis and oxidative stress pathways. Specifically, the LPC (18:0) displays unique immunoregulatory effects characterized by proinflammatory cytokine suppression and antiinflammatory mediator enhancement (31). More importantly, LPC is a potential therapeutic agent for sepsis (32). Previous studies have reported that proinflammatory conditions, including maternal infections, can affect orofacial development (33, 34). High levels of intracellular reactive oxygen species in mouse embryonic palatal cells may eventually result in nsCLP (35). These data further support the decrease in PC (16:0e/22:0) and LPC (18:0), which reduced the antiinflammatory effects during orofacial development. To our knowledge, to date, no studies have associated PC and LPC with fetal orofacial development. Furthermore, glycerophospholipid metabolism was ranked at the top in both untargeted and targeted analyses and was closely related to a group of significant lipid features identified in this study, indicating that PC (16:0e/22:0) and LPC (18:0), may contribute to the developmental biology of lip and palate formations. The free LPC is reacylated by yielding FA, a source of energy, membrane components, and signaling molecules (36). PUFAs regulate the antioxidant signaling pathways and modulate inflammatory processes (37). Omega-3 and omega-6 PUFAs constitute the principal PUFA categories with substantial health implications. Inadequate levels of omega-3 PUFA negatively influence brain function, including behavior, learning, and cognition (38). Omega-3 PUFA-enriched diets prevent phenytoin-induced cleft palate in mid-gestational mice (39), while FA (20:4), an omega-6 PUFA, critically regulates neurodevelopment during pregnancy and early childhood, demonstrating the importance of FA balance throughout development (40).

This study has some limitations. ML-based lipidomics analysis was built and validated by the discovery, validation, and additional validation cohorts, in which all pregnant women from a single center were included. The suitability of this approach for prenatal lipid diagnosis in other populations requires further investigation. Therefore, multicenter validation studies with large cohorts are required to confirm the clinical utility of these lipid biomarkers before clinical implementation. In humans, palatogenesis is initiated in the early sixth week of gestation and palatal fusion is completed by the twelfth week of gestation (41). To explore early prenatal biomarkers, we established a prospective birth cohort study. Due to the low incidence of nsCLP, a small number of specimens were included. A third limitation was the lack of a robust relationship among the panel of 3 lipids involved in orafacial development. As the mechanism of abnormal lipid metabolism in nsCLP is very complex, whether it is caused by genetic factors or environmental factors needs to be further studied. Further studies are needed to fully evaluate the mechanisms underlying the 3 lipid features associated with nsCLP development.

In conclusion, this study provides an approach for the identification of fetal nsCLPs based on untargeted and targeted lipidomics combined with the ML algorithm. These lipid biomarkers and NB-based ML algorithm exhibited strong accuracy in identifying fetal nsCLP, highlighting their value in prenatal diagnosis. The advantages of this diagnostic strategy include minimally invasive sampling, rapid analytical efficiency, and reliable accuracy. Furthermore, our study demonstrated the feasibility, strengths, and promising potential of a strategy combining lipidomics and ML algorithms for identifying disease-related biomarkers.

## Methods

### Sex as a biological variable

Sex was not considered as a biological variable. For discovery, validation and additional validation cohorts, the serum samples from pregnant women were tested.

### Study population and sample collection

**Figure 6:**
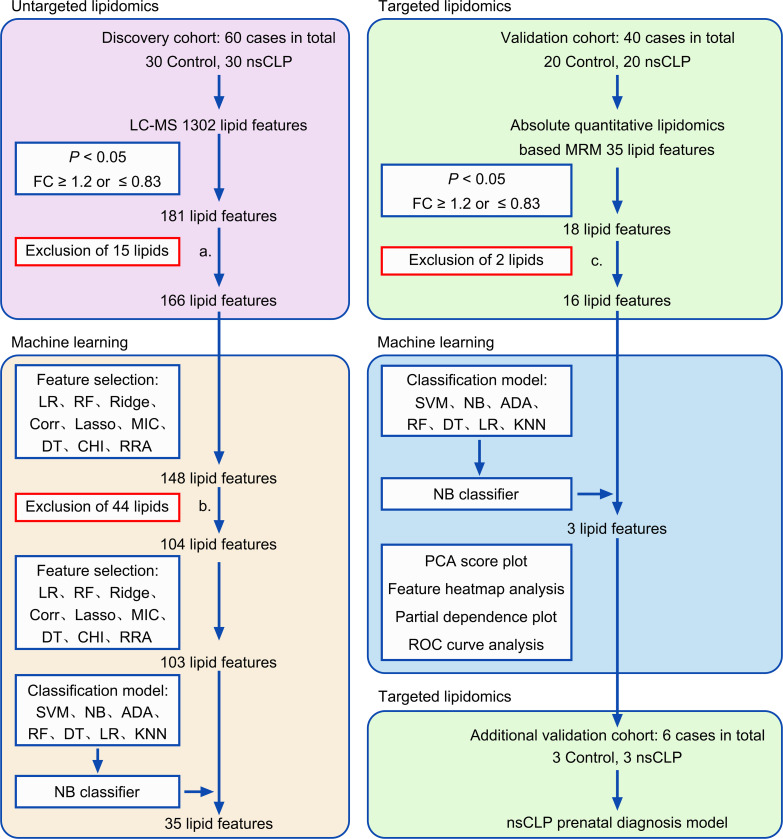
Study design using untargeted and targeted lipidomics analysis combined with machine learning. Untargeted lipidomics: LC-MS–based untargeted lipidomics were initially used for serum lipid profiling in a discovery cohort, and the results were processed using ensemble feature selection methods and 7 classification models to screen for potential lipid biomarkers. The top 35 lipid features were selected as potential biomarkers for further validation using targeted lipidomics. NB classifier: An optimal classification model was constructed using untargeted and targeted lipidomic data. Targeted lipidomics: Absolute quantitative lipidomics-based MRM was used for serum lipid profiling in the validation and additional validation cohort, and the results were processed using multivariate analysis. (a) Fifteen lipid features were excluded as they could not be validated in the future targeted lipidomics. (b) Forty-four lipid features were excluded from isomers. (c) These 2 lipid features were excluded because the expression trend was opposite in untargeted lipidomics.

Fifty-three pregnant women carrying fetuses with nsCLP and 53 pregnant women carrying normal fetuses for discovery, validation, and additional validation cohorts were obtained from Shengjing Hospital of China Medical University. The discovery cohort comprised 30 pregnant women carrying fetuses with nsCLP and 30 pregnant women carrying normal fetuses. In the discovery cohort, lipids were profiled using untargeted lipidomics. The validation cohort included 20 pregnant women with nsCLP and 20 pregnant women with normal fetuses. For lipid biomarker validation, targeted lipidomics were performed in the validation cohort. Serum samples in the discovery and validation cohort were obtained at 22–26 weeks of pregnancy. The additional validation cohort included 3 pregnant women with fetuses with nsCLP and 3 pregnant women with normal fetuses. Serum samples in the additional validation cohort were obtained at 16–17 weeks of pregnancy. Serum samples for lipidomic analysis were obtained from routine venipuncture after ultrasound examinations in an ongoing birth cohort study, which was reported in a previous study (42). Blood was centrifuged within 30 minutes of collection, and the isolated sera were stored at –80°C until analysis. The study workflow is presented in Figure 6. All participants included in this study were matched for gestational age, maternal age, and BMI (Table 3). There was no statistical difference in the sex of the fetuses (Supplemental Table 3). Gynecologists and fetal ultrasonologists at our hospital performed evaluations to ensure accuracy of the final diagnosis. Women with multiple pregnancies, pregnancy-related complications, or metabolic diseases were excluded.

### Untargeted lipidomic analysis

#### Lipid sample preparation.

For untargeted lipidomic analysis, 100 μL aliquots of each pregnant woman’s serum sample were mixed with 240 μL precooled methanol, 800 μL methyl tert-butyl ether (MTBE), and 200 μL water; incubated for 20 minutes; and centrifuged for 15 minutes at 8,000*g*. The the upper lipid layer was obtained and then dried by nitrogen gas and mixed with 240 μL isopropanol, followed by centrifugation for 10 minutes at 8,000*g* under the optimized column temperature at 10°C. For untargeted and targeted lipidomics, equivalent aliquots of each serum sample from the control and nsCLP groups were mixed as QC samples and used to monitor the repeatability of sample preparation and stability of the lipidomic system throughout the experiment.

#### Chromatographic and mass spectrometric conditions.

Untargeted lipidomic analysis was performed using an ultra-high-performance liquid chromatography system (UHPLC; Nexera LC-30A) equipped with a mass spectrometer (Q Exactive Plus; Thermo Scientific) at Shanghai Applied Protein Technology Co. Ltd. Reverse-phase chromatography was selected for LC separation using a CSH C18 column (2.1 mm × 100 mm, 1.7 μm, Waters). The column temperature was set at 45°C, and flow rate was set at 300 μL/min. The aqueous components comprised 10 mM ammonium acetate in acetonitrile/water (6:4, v/v) as the mobile phase A and 10 mM ammonium acetate in acetonitrile/isopropanol (1:9, v/v) as the mobile phase B. The gradient elution program was as follows: from 0 to 2 minutes, B was maintained at 30%; from 2.1 to 25 minutes, B linearly changed from 30% to 100%; and from 25.1 to 30 minutes, B was maintained at 30%. During the entire analysis process, the samples were placed in an autosampler at 10°C. In order to avoid the influence caused by fluctuations in the instrument’s detection signals, continuous analysis of the samples was carried out in a random order. In the sample queue, a QC sample was placed after every 8 experimental samples, which was used to monitor and evaluate the stability of the system as well as the reliability of the experimental data. Lipid species were measured by electrospray ionization mass spectrometry in the positive and negative ion modes. If a lipid is identified in both modes, the mode with high signal strength and stable ionization efficiency was selected for quantification. Experiments in the positive ion mode were performed as follows: heater temperature, 300°C; sheath gas glow rate, 45 AU; aux gas flow rate, 15 AU; sweep gas flow rate, 1 AU; spray voltage, 3.0 KV; capillary temp, 350°C; and S-Lens RF level, 50%. The MS scan ranged from 200 to 1,800. The negative ion mode was performed using the following parameters: heater temperature, 300°C; sheath gas flow rate, 45 AU; aux gas flow rate, 15 AU; sweep gas flow rate, 1 AU; spray voltage, 2.5 KV; capillary temp, 350°C; and S-Lens RF level, 60%. The MS scan ranged from 250 to 1,800. Peak matching, retention time alignment, and peak area extraction were performed using the LipidSearch software version 4.1 (Thermo Scientific). The major parameters were set as follows: precursor tolerance, 5 ppm; product tolerance, 5 ppm; and product ion threshold, 5%. LipidSearch contains data on more than 30 lipid classes, with information on more than 1,700,000 ion fragments. For the data extracted by LipidSearch, missing values for more than 50% of lipid molecules were deleted.

### Targeted lipidomic analysis

#### Lipid sample preparation.

For targeted lipidomic analysis, 50 μL aliquots of each pregnant woman’s serum sample were mixed with 200 μL methanol, 20 μL internal lipid standards, and 800 μL MTBE. The mixture was adequately vortexed, sonicated for 20 minutes at 4°C, and subsequently kept for 30 minutes in room temperature. Subsequently, 200 μL MS-grade water was added. After vortex mixing for 20 seconds, the mixture was vortexed and centrifuged at 18,800*g* at 4°C for 15 minutes. The upper organic solvent layer was obtained and dried under the nitrogen atmosphere. The residue was reconstituted with 200 μL of 90% isopropanol/acetonitrile mixture and centrifuged at 18,800*g* at 4°C for 15 minutes. The supernatant was transferred to an autosampler vial and used for subsequent detection.

#### Chromatographic and mass spectrometric conditions.

UHPLC coupled with triple-quadrupole linear ion-trap tandem mass spectrometry was used for targeted lipidomic analyses. Analysis was performed using a UHPLC system (Nexera LC-30A, Shimadzu) and separated on HILIC (Phenomenex, Luna NH2, 2.0 mm × 100 mm, 3 μm) and C18 (Phenomenex, Kinetex C18, 2.1 mm × 100 mm, 2.6 μm) columns. The HILIC NH_2_ column temperature was maintained at 40°C, and flow rate was set at 0.4 mL/min. The aqueous components comprised 10 mM ammonium acetate in methanol/acetonitrile (5:5, v/v) as the mobile phase A and 10 mM ammonium acetate in acetonitrile/water (5:5, v/v) as the mobile phase B. The gradient program was performed using the following parameters: 0–3 minutes, 3% B; 3–13 minutes, 3% B to 100% B; 13–17 minutes, 100% B; and 17–22 minutes, 3% B. The C18 column temperature was set at 40°C and at a flow rate of 0.35 mL/min. The aqueous component comprised 5 mM ammonium acetate in acetonitrile/water (7:3, v/v) as mobile phase A and IPA solution (isopropanol + 5 mM ammonium acetate) as mobile phase B. The gradient duration program was as follows: from 0 to 5 minutes, 20% B was changed to 60% B; from 5 to 13 minutes, 60% B was changed to 100% B; and from 13 to 17 minutes, B was maintained at 20%. The samples were placed at 10°C during the whole analysis process. 6500+QTRAP (AB SCIEX) was used in positive and negative switch modes. ESI was performed using the following parameters: source temperature, 400°C; ion source gas 1, 50; ion source gas 2, 55; curtain gas, 35; and ionSpray voltage, +5,500 V or –4,500 V in positive or negative modes, respectively. The MRM method was used for mass spectrometry quantitative data acquisition. Sciex OS software was used for the peak extraction of raw MRM data to obtain each lipid and ratio of peak area to internal standard peak area. The content of each lipid was subsequently calculated.

### ML

#### Feature preliminary filtering method.

We applied the 3-sigma principle to detect outliers in all data. For all identified outliers, we used the KNN algorithm with a neighbor count of 5 to fill in the missing values and outliers. Moreover, we performed a log_2_ transformation on all data to reduce the scale disparities among features and to help achieve a more stable and comparable distribution for subsequent statistical analyses and ML modeling. Thereafter, we performed an initial statistical filtering step to focus on differentially expressed lipids. Specifically, we applied a significance level of *P* < 0.05 and FC thresholds (FC ≥ 1.2 or ≤ 0.83) based on commonly accepted standards and empirical evidence (43, 44). This approach helps reduce noise, lower computational complexity, and ensure that subsequent ML analyses prioritize lipid features with greater biological relevance. These criteria were used to generate a subset of features that satisfied the filtering conditions.

#### Ensemble feature selection.

To avoid the overfitting and variability in credibility caused by the use of a single method, we used the ensemble-FS method. Specifically, datasets were represented using the following formula: *D*_M × N_ = *X*_1_, *X*_2_... *X_i_*..., *X_M_*^,^ *X_i_ =* (*x_i_*^(1)^, *x_i_*^(2)^..., *x_i_* ^(*j*)^..., *x_i_* ^(*N*)^)*^T^*, where *M* denotes the number of samples and *N* denotes the number of features, *X_i_* represents the expression values of all features for the *i*-th sample, and *x_i_*^(1)^ represents the expression value matrix of the *j*-th feature in the *i*-th sample. The objective of the feature selection method was to construct a feature evaluation model, represented as *EV*(*), through which the data are evaluated to produce a subspace X_i_^*^ = (*x_i_*^(1)^, *x_i_* ^(2)^..., *x_i_*^(*s*)^)*^T^* (*S* < *N*) of the original features. The asterisk in *EV*(*) denotes a placeholder, indicating that this evaluation function, *EV*, can operate on any feature or feature subset within our dataset. The letter *S* represents the number of selected features after dimensionality reduction, which is smaller than the original feature dimension *N*. We employed 8 ML algorithms that can output feature rankings to form a multidimensional feature evaluator. Each algorithm was used to independently evaluate all lipid features, resulting in a feature-ranking list. These methods include L1 regularization (Lasso), L2 regularization (Ridge), RF, linear regression (LR), correlation, DT, χ^2^ test, and maximal information coefficient. To ensure that different feature selection algorithms obtain a reasonable number of features, the parameter settings were as follows. For the Lasso and Ridge methods, the penalty coefficient was set to 0.01, and the stopping criterion was set to 0.001. For the DT and RF methods, the Gini coefficient was used as the criterion for node splitting. The maximum depth was set to none, min_samples_split was set to 2, and min_samples_leaf was set to 1. The other 4 methods used the default parameters in sklearn 1.2.2.

The RRA approach was used to combine the evaluation results from multiple feature evaluators and generate a unified ranking list. The features are ranked by scores, resulting in a final feature ranking. In our previous study, we combined this method with a genetic algorithm for feature-selection tasks using microarray data (45). We subsequently input the corresponding feature-ranking results into the RRA algorithm to obtain a unified feature ranking. This method detected lipid features that were ranked higher than expected under the null hypothesis of irrelevance and assigned a significance score to each feature.

### Classification model

To identify the optimal ML model for distinguishing between nsCLP and control samples using lipid features, 7 different classifier models were constructed as follows: SVM, NB, RF, DT, LR, ADA, and KNN. All the classification models also used the default parameters in sklearn 1.2.2. In both untargeted and targeted lipidomics, the samples were stratified into the training and testing sets in a 4:1 manner, and the classifier models were subsequently trained using a 5-fold cross-validation method.

### Statistics

The ML experiments were conducted on a Windows 11 system equipped with an Intel Core i7-12700H CPU, 32 GB of RAM, and a GTX 1060 GPU. For all ML experiments, to ensure the reliability of results, a 5-fold cross-validation method was employed, and the average value of the results was considered as the final result. The implementation of the ensemble-FS was based on scikit-learn version 1.1.2. The OPLS-DA model and permutation test of the OPLS-DA model were performed using SIMCA-P software (version 14.1; Umetrics). Pathway enrichment analysis was conducted using LIPEA (https://hyperlipea.org/home) with the Kyoto Encyclopedia of Genes and Genomes database (https://www.genome.jp/kegg/), and results are represented by a bubble plot (Figure 3D and Figure 4C). PCA and receiver operating characteristic (ROC) curves were used to assess the predictive performance of potential lipid biomarkers using Python 3.9. Two groups of data with Gaussian distributions were compared using 2-tailed Student’s *t* tests. Otherwise, nonparametric Mann-Whitney tests were used. A *P* value of less than 0.05 was considered significant. Prism Graphpad software (version 7.0) and SPSS 22.0 were used for statistical analysis.

### Study approval

All clinical studies were conducted according to the Declaration of Helsinki principles. Written informed consent was obtained from all pregnant women, and the study was approved by the Ethics Committee of the Shengjing Hospital (approval 2017PS264K).

### Data availability

Details regarding lipid features at every step of the ML approach are available in the Supplemental Data. Values for all data points shown in graphs are reported in the Supporting Data Values file. The raw data supporting the conclusions of this article will be made available from the corresponding author upon request.

## Author contributions

SJ, WX, W Li, and ZY designed the study. SJ and CY contributed to data analysis. YD prepared serum samples. WX and W Li performed ML experiments. SJ and WX wrote the first draft of the paper. W Luo, HG, XW, WM, DL, SC, and YB contributed to lipidomic analysis. The authorship order among co–first authors (SJ first and WX second) was based on their contributions to drafting the manuscript. All authors read and approved the final manuscript.

## Supplementary Material

Supplemental data

Supporting data values

## Figures and Tables

**Table 3:**
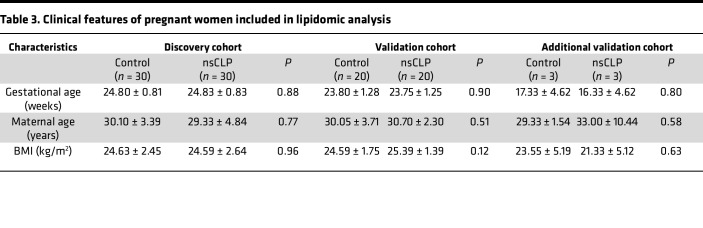
Clinical features of pregnant women included in lipidomic analysis

**Table 2:**
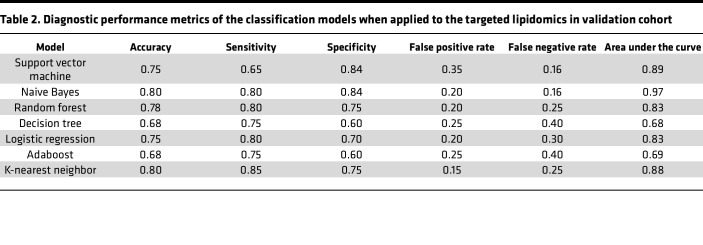
Diagnostic performance metrics of the classification models when applied to the targeted lipidomics in validation cohort

**Table 1:**
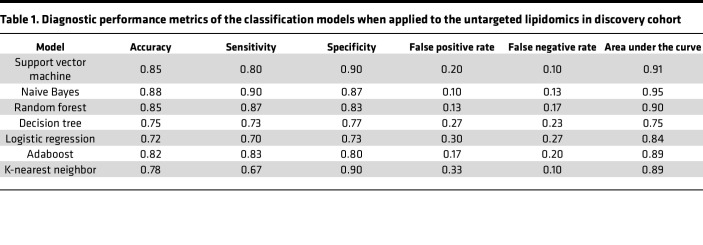
Diagnostic performance metrics of the classification models when applied to the untargeted lipidomics in discovery cohort

## Version 1 05/08/2025

Electronic publication
