# Supplementary material for: Combining lipidomics and machine learning to identify lipid biomarkers for nonsyndromic cleft lip with palate
Source: JCI Insight. 2025 May 8;10(9):e186629. doi: 10.1172/jci.insight.186629 (PMC12128965; doi:10.1172/jci.insight.186629)
Supplement: Supplemental data [file jciinsight-10-186629-s132.pdf]

Title: Combining lipidomics and machine learning to identify lipid biomarkers for nonsyndromic cleft lip with palate

Supplemental Figure 1. QC RSD distribution of quality control samples in the lipidomic data.

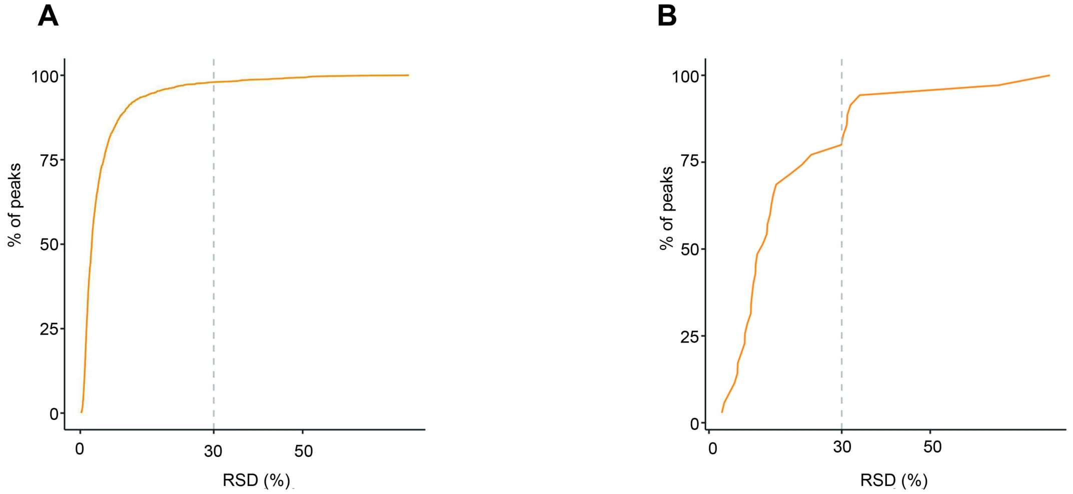

(A) QC RSD distribution of quality control samples in the untargeted lipidomics. **(B)** QC RSD distribution of quality control samples in the targeted lipidomics.

Supplemental Figure 2. Training curve analysis of the six different classification models, including SVM, RF, DT, LR, ADA and KNN.

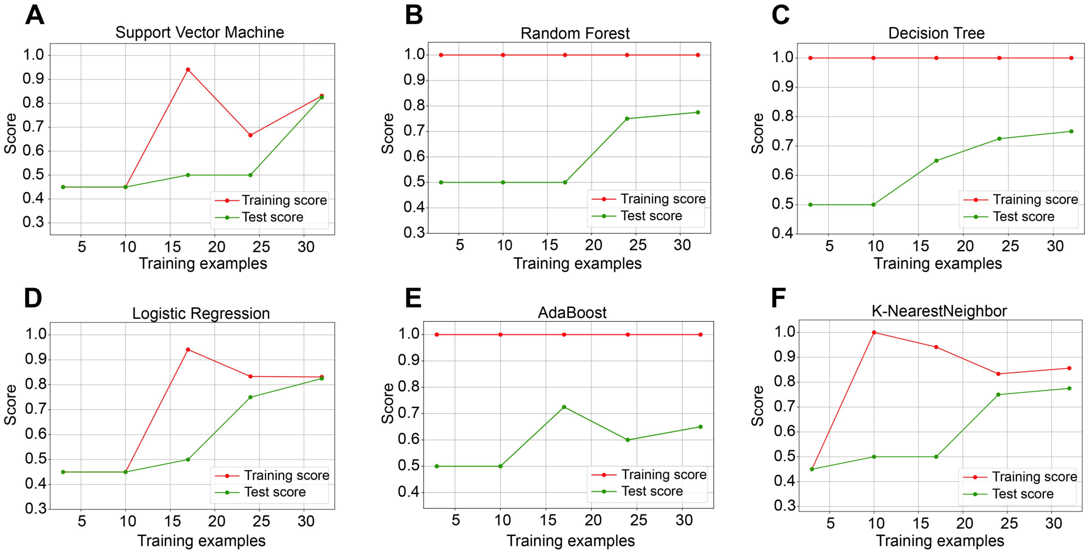

(A) Learning curve analysis of the SVM classifier. **(B)** Learning curve analysis of the RF classifier. **(C)** Learning curve analysis of the DT classifier. **(D)** Learning curve analysis of the LR classifier. **(E)** Learning curve analysis of the ADA classifier. **(F)** Learning curve analysis of the KNN classifier.

Supplemental Figure 3. Relative expression of the three lipid features in the additional validation phase.

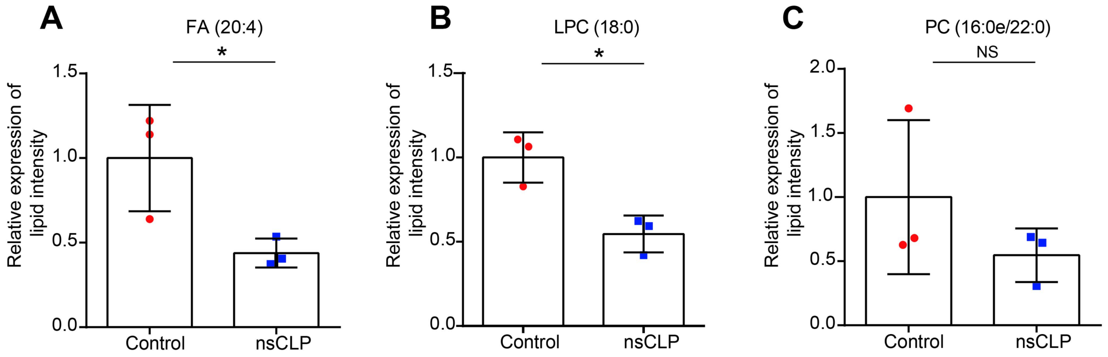

(A) Relative expression of the FA(20:4) in nsCLP compared with control group (data are presented as mean ± SD, by unpaired t-test; n=3, each group). **(B)** Relative expression of the LPC(18:0) in nsCLP compared with control group (data are presented as mean ± SD, by unpaired t-test; n=3, each group). **(C)** Relative expression of the PC(16:0e/22:0) in nsCLP compared with control group (data are presented as mean ± SD, by unpaired t-test; n=3, each group). *$P < 0.05$; NS, $P \geq 0.05$.

Supplemental Table 1. Fifteen pathways enriched by 35 dysregulated lipid features.

Pathway name	Pathway lipids	Converted lipids (number)	Converted lipids (percentage)	Converted lipids (list)	*P*-value	Benjamini correction	Bonferroni correction
Glycerophospholipid metabolism	26	7	58.33333333	C04438, C05973, C04230, C00157, C04233, C00350, C03819	1.87862E-07	2.81793E-06	2.81793E-06
Ferroptosis	11	3	25	C21480, C21481, C21484	0.001298164	0.00973623	0.01947246
Choline metabolism in cancer	5	2	16.66666667	C04230, C00157	0.004465587	0.022327933	0.0669838
Retrograde endocannabinoid signaling	8	2	16.66666667	C00157, C00350	0.012039813	0.045149299	0.180597194
Pathogenic Escherichia coli infection	1	1	8.333333333	C00350	0.02247191	0.06741573	0.337078652
Glycosylphosphatidylinositol (GPI)-anchor biosynthesis	3	1	8.333333333	C00350	0.066033129	0.123812117	0.990496933
Autophagy - other	3	1	8.333333333	C00350	0.066033129	0.123812117	0.990496933
Kaposi's sarcoma-associated herpesvirus infection	3	1	8.333333333	C00350	0.066033129	0.123812117	0.990496933
Autophagy - animal	4	1	8.333333333	C00350	0.087139725	0.130709587	1
Necroptosis	4	1	8.333333333	C00550	0.087139725	0.130709587	1
Sphingolipid signaling pathway	9	1	8.333333333	C00550	0.186265297	0.253998133	1
Sphingolipid metabolism	21	1	8.333333333	C00550	0.385271614	0.472457694	1
alpha-Linolenic acid metabolism	23	1	8.333333333	C00157	0.4137219	0.472457694	1
Linoleic acid metabolism	25	1	8.333333333	C00157	0.440960514	0.472457694	1
Arachidonic acid metabolism	75	1	8.333333333	C00157	0.840651573	0.840651573	1

Supplemental Table 2. Ten pathways enriched by 16 dysregulated lipid features.

Pathway name	Pathway lipids	Converted lipids (number)	Converted lipids (percentage)	Converted lipids (list)	*P*-value	Benjamini correction	Bonferroni correction
Glycerophospholipid metabolism	26	6	66.66666667	C04230, C03819, C00157, C05973, C04233, C04438	5.41132E-07	5.41132E-06	5.41132E-06
Choline metabolism in cancer	5	2	22.22222222	C04230, C00157	0.002436551	0.012182753	0.024365507
Necroptosis	4	1	11.11111111	C00550	0.065551286	0.218504285	0.655512856
Retrograde endocannabinoid signaling	8	1	11.11111111	C00157	0.127254856	0.284206144	1
Sphingolipid signaling pathway	9	1	11.11111111	C00550	0.142103072	0.284206144	1
Ferroptosis	11	1	11.11111111	C21484	0.171127574	0.285212623	1
Sphingolipid metabolism	21	1	11.11111111	C00550	0.303564456	0.390034524	1
alpha-Linolenic acid metabolism	23	1	11.11111111	C00157	0.327670026	0.390034524	1
Linoleic acid metabolism	25	1	11.11111111	C00157	0.351031071	0.390034524	1
Arachidonic acid metabolism	75	1	11.11111111	C00157	0.74459572	0.74459572	1

Supplemental Table 3. Sex features of foetuses included in lipidomic analysis.

Foetuses sex	Discovery cohort			Validation cohort			Additional validation cohort		
	Control (n = 30)	nsCLP (n = 30)	*P*	Control (n = 20)	nsCLP (n = 20)	*P*	Control (n = 3)	nsCLP (n = 3)	*P*
male	13	16	0.44	11	8	0.34	1	2	1.00
female	17	14		9	12		2	1	

Supplemental Data. Details of lipid features at every step of the machine learning approach.

untargeted lipidomics (n=1302)	FC ≥ 1.2 or ≤ 0.83, *P* < 0.05 (n=181)	15 lipids were excluded for could not be validated in the targeted lipidomics (n=166)	feature selection (n=148)	44 lipids were excluded for isomers (n=104)	feature selection (n=103)	targeted lipidomics (n=35)	FC ≥ 1.2 or ≤ 0.83, *P* < 0.05 (n=16)	panel of lipid biomarkers (n=3)
M524T237_POS	M520T154_POS	M520T154_POS	M943T993_NEG	M943T993_NEG	M524T237_POS	M524T237_POS	M802T858_POS	M303T298_NEG (FA[20:4])
M546T256_POS	M450T167_POS	M704T329_POS	M480T204_POS	M480T204_POS	M546T256_POS	M546T256_POS	M303T298_NEG	M524T237_POS (LPC[18:0])
M524T256_POS	M704T329_POS	M661T226_POS	M482T213_POS	M524T237_POS	M538T308_POS	M538T308_POS	M327T272_NEG	M849T948_NEG (PC[16:0e/22:0])
M496T169_POS	M424T153_POS	M606T230_POS	M524T237_POS	M580T219_POS	M480T204_POS	M480T204_POS	M524T237_POS	
M496T182_POS	M661T226_POS	M772T519_POS	M508T291_POS	M965T1019_NEG	M510T214_POS	M510T214_POS	M548T205_POS	
M480T272_NEG	M400T179_POS	M595T126_NEG	M580T219_POS	M805T566_POS	M534T238_POS	M534T238_POS	M504T272_POS	
M522T191_POS	M606T230_POS	M564T155_NEG	M965T1019_NEG	M802T858_POS	M327T272_NEG	M327T272_NEG	M599T197_NEG	
M452T192_NEG	M426T189_POS	M746T519_POS	M805T566_POS	M534T238_POS	M802T858_POS	M802T858_POS	M538T308_POS	
M520T154_POS	M772T519_POS	M542T154_POS	M802T858_POS	M781T637_POS	M943T993_NEG	M943T993_NEG	M480T204_POS	
M745T707_POS	M595T126_NEG	M580T219_POS	M568T238_NEG	M969T1478_POS	M744T527_NEG	M744T527_NEG	M510T214_POS	
M720T663_NEG	M452T201_POS	M468T137_POS	M508T223_POS	M869T846_POS	M781T637_POS	M781T637_POS	M466T228_NEG	
M788T838_POS	M564T155_NEG	M524T237_POS	M534T238_POS	M546T256_POS	M466T228_NEG	M466T228_NEG	M506T214_POS	
M826T614_NEG	M746T519_POS	M546T256_POS	M510T304_POS	M751T726_POS	M504T272_POS	M504T272_POS	M536T314_POS	
M825T615_NEG	M542T154_POS	M524T256_POS	M781T637_POS	M947T1201_POS	M506T214_POS	M506T214_POS	M849T948_NEG	
M947T1201_POS	M580T219_POS	M496T169_POS	M969T1478_POS	M744T527_NEG	M704T329_POS	M704T329_POS	M536T224_POS	
M667T1327_POS	M380T250_POS	M496T182_POS	M869T846_POS	M773T765_POS	M606T474_POS	M606T474_POS	M815T710_NEG	
M815T710_NEG	M346T193_POS	M480T272_NEG	M524T256_POS	M937T1265_POS	M544T191_POS	M544T191_POS		
M773T765_POS	M454T261_POS	M522T191_POS	M496T169_POS	M510T214_POS	M947T1201_POS	M947T1201_POS		
M747T687_NEG	M468T137_POS	M452T192_NEG	M546T256_POS	M536T314_POS	M580T219_POS	M580T219_POS		
M816T908_POS	M524T237_POS	M745T707_POS	M751T726_POS	M685T759_POS	M303T298_NEG	M303T298_NEG		
M793T678_POS	M546T256_POS	M720T663_NEG	M947T1201_POS	M504T272_POS	M599T197_NEG	M599T197_NEG		

M814T846_POS	M524T256_POS	M825T615_NEG	M744T527_NEG	M746T519_POS	M661T226_POS	M661T226_POS
M482T213_POS	M496T169_POS	M947T1201_POS	M773T765_POS	M1007T1386_POS	M705T710_POS	M705T710_POS
M480T204_POS	M496T182_POS	M815T710_NEG	M937T1265_POS	M552T166_NEG	M536T314_POS	M536T314_POS
M568T238_NEG	M480T272_NEG	M773T765_POS	M510T214_POS	M466T228_NEG	M879T679_NEG	M879T679_NEG
M568T258_NEG	M522T191_POS	M793T678_POS	M546T237_POS	M879T679_NEG	M965T1019_NE G	M965T1019_NEG
M508T291_POS	M452T192_NEG	M482T213_POS	M536T314_POS	M847T971_POS	M849T948_NEG	M849T948_NEG
M538T308_POS	M745T707_POS	M480T204_POS	M685T759_POS	M884T613_NEG	M536T224_POS	M536T224_POS
M508T223_POS	M720T663_NEG	M568T238_NEG	M504T272_POS	M849T948_NEG	M767T580_POS	M767T580_POS
M506T257_POS	M826T614_NEG	M568T258_NEG	M746T519_POS	M943T1081_POS	M815T710_NEG	M815T710_NEG
M546T237_POS	M825T615_NEG	M508T291_POS	M1007T1386_POS	M564T155_NEG	M871T920_POS	M871T920_POS
M510T304_POS	M947T1201_POS	M538T308_POS	M552T166_NEG	M747T663_NEG	M937T1265_POS	M937T1265_POS
M506T214_POS	M815T710_NEG	M508T223_POS	M466T228_NEG	M578T375_POS	M773T765_POS	M773T765_POS
M534T238_POS	M773T765_POS	M506T257_POS	M879T679_NEG	M772T519_POS	M548T205_POS	M548T205_POS
M518T182_POS	M793T678_POS	M546T237_POS	M847T971_POS	M805T807_POS	M919T1142_POS	M919T1142_POS
M536T314_POS	M482T213_POS	M510T304_POS	M518T182_POS	M850T855_POS	M720T663_NEG	
M518T169_POS	M480T204_POS	M506T214_POS	M884T613_NEG	M705T772_POS	M943T1081_POS	
M582T309_NEG	M568T238_NEG	M534T238_POS	M849T948_NEG	M909T1198_POS	M619T396_POS	
M482T272_POS	M568T258_NEG	M518T182_POS	M943T1081_POS	M999T1236_POS	M903T953_POS	
M504T272_POS	M508T291_POS	M536T314_POS	M564T155_NEG	M538T308_POS	M805T807_POS	
M554T214_NEG	M538T308_POS	M518T169_POS	M747T663_NEG	M568T169_POS	M909T1198_POS	
M550T266_POS	M508T223_POS	M582T309_NEG	M578T375_POS	M859T1342_POS	M600T246_POS	
M510T214_POS	M506T257_POS	M482T272_POS	M772T519_POS	M614T168_NEG	M578T375_POS	
M540T182_NEG	M546T237_POS	M504T272_POS	M496T182_POS	M792T707_NEG	M699T718_NEG	
M536T224_POS	M510T304_POS	M554T214_NEG	M805T807_POS	M506T214_POS	M935T1236_POS	
M1036T182_NE	M506T214_POS	M550T266_POS	M850T855_POS	M767T580_POS	M755T776_POS	

G					
M303T298_NEG	M534T238_POS	M510T214_POS	M705T772_POS	M784T797_POS	M595T126_NEG
M532T214_POS	M518T182_POS	M540T182_NEG	M909T1198_POS	M815T710_NEG	M784T797_POS
M548T205_POS	M536T314_POS	M536T224_POS	M999T1236_POS	M618T746_POS	M805T566_POS
M478T182_POS	M518T169_POS	M303T298_NEG	M538T308_POS	M699T718_NEG	M847T971_POS
M454T192_POS	M582T309_NEG	M532T214_POS	M568T169_POS	M921T1125_POS	M1013T1496_PO S
M599T197_NEG	M482T272_POS	M548T205_POS	M582T309_NEG	M945T1486_POS	M580T473_POS
M466T228_NEG	M504T272_POS	M478T182_POS	M859T1342_POS	M871T920_POS	M909T1348_POS
M552T369_POS	M554T214_NEG	M454T192_POS	M614T168_NEG	M595T126_NEG	M945T1486_POS
M592T205_NEG	M550T266_POS	M599T197_NEG	M792T707_NEG	M600T246_POS	M909T1227_POS
M596T369_NEG	M510T214_POS	M466T228_NEG	M482T272_POS	M777T746_POS	M899T992_POS
M544T191_POS	M540T182_NEG	M552T369_POS	M773T805_POS	M661T226_POS	M757T842_POS
M594T267_NEG	M536T224_POS	M592T205_NEG	M542T154_POS	M783T677_POS	M859T1342_POS
M476T192_POS	M1036T182_NEG	M596T369_NEG	M506T214_POS	M548T205_POS	M586T128_NEG
M540T169_NEG	M303T298_NEG	M544T191_POS	M767T580_POS	M734T591_NEG	M823T552_NEG
M538T421_POS	M532T214_POS	M594T267_NEG	M520T154_POS	M720T663_NEG	M468T137_POS
M810T840_POS	M548T205_POS	M476T192_POS	M784T797_POS	M705T710_POS	M747T663_NEG
M327T272_NEG	M478T182_POS	M540T169_NEG	M518T169_POS	M935T1200_POS	M823T572_NEG
M450T167_POS	M454T192_POS	M538T421_POS	M532T214_POS	M801T572_1_POS	M701T719_POS
M606T474_POS	M599T197_NEG	M327T272_NEG	M815T710_NEG	M572T185_POS	M751T726_POS
M704T329_POS	M466T228_NEG	M606T474_POS	M618T746_POS	M823T572_NEG	M526T156_NEG
M508T166_POS	M552T369_POS	M508T166_POS	M699T718_NEG	M757T842_POS	M608T562_POS
M424T153_POS	M592T205_NEG	M566T190_NEG	M921T1125_POS	M909T1227_POS	M825T615_NEG
M566T190_NEG	M596T369_NEG	M797T821_NEG	M945T1486_POS	M793T607_POS	M614T168_NEG
M797T821_NEG	M544T191_POS	M578T375_POS	M478T182_POS	M825T615_NEG	M705T772_POS

M578T375_POS	M594T267_NEG	M552T166_NEG	M568T258_NEG	M935T1236_POS	M1007T1386_POS
M552T166_NEG	M476T192_POS	M580T473_POS	M612T141_NEG	M611T792_POS	M969T1478_POS
M661T226_POS	M540T169_NEG	M704T665_POS	M506T257_POS	M303T298_NEG	M850T855_POS
M400T179_POS	M538T421_POS	M937T1265_POS	M765T644_POS	M327T272_NEG	M777T746_POS
M606T230_POS	M327T272_NEG	M548T191_POS	M871T920_POS	M468T137_POS	M792T707_NEG
M300T170_POS	M606T474_POS	M662T533_POS	M595T126_NEG	M526T156_NEG	M568T169_POS
M426T189_POS	M508T166_POS	M689T997_POS	M600T246_POS	M536T224_POS	M893T1124_POS
M580T473_POS	M566T190_NEG	M792T707_NEG	M777T746_POS	M544T191_POS	M753T542_POS
M772T519_POS	M797T821_NEG	M919T1142_POS	M661T226_POS	M586T128_NEG	M921T1125_POS
M595T126_NEG	M578T375_POS	M482T156_POS	M783T677_POS	M580T473_POS	M734T591_NEG
M704T665_POS	M552T166_NEG	M869T846_POS	M548T205_POS	M608T562_POS	M746T519_POS
M881T1150_POS	M300T170_POS	M600T246_POS	M734T591_NEG	M606T474_POS	M935T1200_POS
M452T201_POS	M580T473_POS	M795T652_POS	M720T663_NEG	M454T192_POS	M884T613_NEG
M864T1150_1_POS	M704T665_POS	M568T169_POS	M540T182_NEG	M599T197_NEG	M662T533_POS
M564T155_NEG	M881T1150_POS	M611T792_POS	M705T710_POS	M823T552_NEG	M793T678_POS
M937T1265_POS	M864T1150_1_POS	M999T1236_POS	M935T1200_POS	M606T230_POS	M552T166_NEG
M643T1385_POS	M937T1265_POS	M751T726_POS	M801T572_1_POS	M753T542_POS	M795T652_POS
M746T519_POS	M548T191_POS	M973T1510_POS	M548T191_POS	M795T652_POS	M801T572_1_POS
M548T191_POS	M662T533_POS	M857T969_POS	M572T185_POS	M805T948_POS	M973T1510_POS
M662T533_POS	M689T997_POS	M884T613_NEG	M823T572_NEG	M793T678_POS	M783T677_POS
M689T997_POS	M792T707_NEG	M943T993_NEG	M757T842_POS	M845T905_POS	M572T185_POS
M792T707_NEG	M919T1142_POS	M893T1124_POS	M909T1227_POS	M899T992_POS	M564T155_NEG

M919T1142_POS	M482T156_POS	M731T788_POS	M570T169_POS	M903T953_POS	M845T905_POS
M482T156_POS	M869T846_POS	M969T1478_POS	M793T607_POS	M701T719_POS	M999T1236_POS
M869T846_POS	M600T246_POS	M618T746_POS	M825T615_NEG	M797T821_NEG	M805T948_POS
M600T246_POS	M795T652_POS	M805T948_POS	M935T1236_POS	M755T776_POS	M797T821_NEG
M542T154_POS	M568T169_POS	M793T607_POS	M611T792_POS	M619T396_POS	M606T230_POS
M795T652_POS	M611T792_POS	M526T156_NEG	M303T298_NEG	M662T533_POS	M454T192_POS
M777T876_POS	M999T1236_POS	M572T185_POS	M327T272_NEG	M704T329_POS	M869T846_POS
M568T169_POS	M751T726_POS	M689T1224_POS	M468T137_POS	M893T1124_POS	M772T519_POS
M611T792_POS	M973T1510_POS	M852T944_POS	M526T156_NEG	M919T1142_POS	M611T792_POS
M999T1236_POS	M857T969_POS	M773T805_POS	M540T169_NEG	M909T1348_POS	M618T746_POS
M751T726_POS	M884T613_NEG	M699T718_NEG	M554T214_NEG	M1013T1496_POS	M793T607_POS
M973T1510_POS	M943T993_NEG	M823T552_NEG	M508T166_POS	M973T1510_POS	
M857T969_POS	M893T1124_POS	M759T736_POS	M522T191_POS		
M884T613_NEG	M731T788_POS	M767T580_POS	M566T190_NEG		
M844T981_POS	M969T1478_POS	M801T572_1_POS	M536T224_POS		
M943T993_NEG	M618T746_POS	M823T572_NEG	M552T369_POS		
M893T1124_POS	M805T948_POS	M907T1163_POS	M596T369_NEG		
M731T788_POS	M793T607_POS	M911T1267_POS	M538T421_POS		
M969T1478_POS	M526T156_NEG	M701T719_POS	M550T266_POS		
M793T757_POS	M572T185_POS	M857T946_POS	M594T267_NEG		
M618T746_POS	M813T1033_POS	M899T992_POS	M592T205_NEG		
M690T612_POS	M689T1224_POS	M753T542_POS	M544T191_POS		
M805T948_POS	M852T944_POS	M777T746_POS	M586T128_NEG		
M580T219_POS	M773T805_POS	M744T527_NEG	M580T473_POS		
M793T607_POS	M699T718_NEG	M685T759_POS	M614T153_NEG		
M526T156_NEG	M823T552_NEG	M705T710_POS	M608T562_POS		

M572T185_POS	M759T736_POS	M779T552_POS	M606T474_POS
M813T1033_POS	M767T580_POS	M725T706_POS	M454T192_POS
M689T1224_POS	M801T572_1_POS	M734T591_NEG	M476T192_POS
M852T944_POS	M823T572_NEG	M570T169_POS	M452T192_NEG
M773T805_POS	M907T1163_POS	M965T1019_NEG	M480T272_NEG
M845T643_POS	M911T1267_POS	M779T572_POS	M599T197_NEG
M699T718_NEG	M701T719_POS	M909T1198_POS	M823T552_NEG
M829T642_POS	M857T946_POS	M859T1342_POS	M606T230_POS
M861T1385_POS	M899T992_POS	M781T615_POS	M753T568_POS
M572T147_POS	M753T542_POS	M945T1486_POS	M753T542_POS
M823T552_NEG	M777T746_POS	M845T905_POS	M779T572_POS
M759T736_POS	M744T527_NEG	M757T842_POS	M795T652_POS
M795T766_POS	M685T759_POS	M608T562_POS	M805T948_POS
M767T580_POS	M705T710_POS	M805T566_POS	M793T678_POS
M801T572_1_POS	M779T552_POS	M612T141_NEG	M845T905_POS
M823T572_NEG	M725T706_POS	M903T953_POS	M857T946_POS
M907T1163_POS	M734T591_NEG	M783T677_POS	M857T969_POS
M911T1267_POS	M570T169_POS	M753T568_POS	M899T992_POS
M701T719_POS	M965T1019_NEG	M747T663_NEG	M903T953_POS
M857T946_POS	M779T572_POS	M1007T1386_POS	M701T719_POS
M899T992_POS	M824T572_NEG	M765T644_POS	M797T821_NEG
M753T542_POS	M909T1198_POS	M847T971_POS	M755T776_POS
M777T746_POS	M859T1342_POS	M805T807_POS	M619T396_POS
M380T250_POS	M781T615_POS	M614T153_NEG	M662T533_POS
M744T527_NEG	M945T1486_POS	M586T128_NEG	M704T329_POS

M834T870_POS	M845T905_POS	M935T1236_POS	M893T1124_POS
M346T193_POS	M757T842_POS	M705T772_POS	M919T1142_POS
M685T759_POS	M608T562_POS	M542T127_POS	M909T1348_POS
M705T710_POS	M805T566_POS	M781T637_POS	M1013T1496_POS
M779T552_POS	M612T141_NEG	M969T1147_POS	M973T1510_POS
M725T706_POS	M903T953_POS	M891T1068_POS	
M734T591_NEG	M783T677_POS	M755T776_POS	
M570T169_POS	M753T568_POS	M614T168_NEG	
M965T1019_NEG	M747T663_NEG	M879T679_NEG	
M779T572_POS	M1007T1386_POS	M849T948_NEG	
M824T572_NEG	M765T644_POS	M933T1167_POS	
M909T1198_POS	M847T971_POS	M909T1227_POS	
M859T1342_POS	M805T807_POS	M912T1022_POS	
M724T741_POS	M614T153_NEG	M921T1125_POS	
M781T615_POS	M586T128_NEG	M850T855_POS	
M945T1486_POS	M935T1236_POS	M871T920_POS	
M845T905_POS	M705T772_POS	M935T1200_POS	
M757T842_POS	M542T127_POS	M619T396_POS	
M671T1435_POS	M781T637_POS	M909T1348_POS	
M608T562_POS	M969T1147_POS	M784T797_POS	
M805T566_POS	M891T1068_POS	M943T1081_POS	
M428T251_POS	M755T776_POS	M1013T1496_POS	
M847T785_POS	M614T168_NEG	M802T858_POS	
M612T141_NEG	M879T679_NEG		
M903T953_POS	M849T948_NEG		

M706T674_POS	M933T1167_POS
M771T810_POS	M909T1227_POS
M771T756_POS	M912T1022_POS
M669T1383_POS	M921T1125_POS
M783T677_POS	M850T855_POS
M753T568_POS	M871T920_POS
M747T663_NEG	M935T1200_POS
M1007T1386_POS	M619T396_POS
M765T644_POS	M909T1348_POS
M847T971_POS	M784T797_POS
M889T1424_POS	M943T1081_POS
M805T807_POS	M1013T1496_POS
M614T153_NEG	M802T858_POS
M454T261_POS	
M586T128_NEG	
M935T1236_POS	
M468T137_POS	
M705T772_POS	
M542T127_POS	
M781T637_POS	
M969T1147_POS	
M891T1068_POS	
M749T688_1_POS	
M755T776_POS	

M614T168_NEG

M879T679_NEG

M700T526_POS

M849T948_NEG

M732T548_NEG

M933T1167_POS

M842T917_POS

M796T806_POS

M909T1227_POS

M912T1022_POS

M726T648_POS

M688T547_POS

M921T1125_POS

M850T855_POS

M871T920_POS

M873T784_POS

M888T981_NEG

M791T832_POS

M865T736_NEG

M935T1200_POS

M619T396_POS

M674T503_POS

M947T1512_POS

M909T1348_POS

M784T797_POS

M943T1081_POS

M1013T1496_PO
S
M802T858_POS
M829T896_POS
M785T770_POS
M731T677_POS
M801T830_POS
M811T746_NEG
M931T654_POS
M917T1071_POS
M881T907_POS
M749T736_POS
M568T141_POS
M873T976_POS
M773T692_NEG
M774T806_POS
M817T765_NEG
M891T1457_POS
M811T653_NEG
M760T775_POS
M845T690_POS
M865T1422_POS
M693T689_POS
M779T787_POS
M993T1089_NE
G

M374T275_POS

M776T831_POS

M735T593_POS

M537T553_POS

M863T1421_POS

M548T153_POS

M819T682_POS

M971T1178_POS

M891T1421_POS

M917T1457_POS

M828T855_POS

M763T770_POS

M718T503_NEG

M943T1458_POS

M1037T1440_PO

S

M775T816_POS

M811T774_POS

M372T135_POS

M1013T976_NE

G

M712T612_POS

M821T737_POS

M745T745_POS

M734T612_NEG

M775T746_NEG

M715T603_NEG

M883T1225_POS

M793T709_POS

M845T755_POS

M290T115_POS

M815T756_NEG

M398T144_POS

M512T137_NEG

M835T1385_POS

M739T643_NEG

M886T651_NEG

M767T707_POS

M679T1090_POS

M717T1290_POS

M995T1148_POS

M570T154_POS

M802T872_POS

M771T712_POS

M977T1288_POS

M636T280_POS

M767T637_POS

M1266T791_POS

M863T681_NEG

M895T906_POS

M961T1419_NE

G

M887T850_NEG
M830T944_POS
M827T843_POS
M993T1084_POS
M769T752_POS
M751T778_POS
M747T801_POS
M915T920_NEG
M843T850_POS
M977T1246_POS
M874T930_NEG
M727T723_POS
M774T831_POS
M853T882_1_PO
S
M907T676_POS
M797T771_POS
M782T774_POS
M823T776_POS
M774T692_NEG
M613T845_POS
M967T1059_POS
M743T698_POS
M897T936_POS
M843T835_POS
M881T953_POS

M824T872_POS

M881T1441_POS

M720T705_POS

M749T810_POS

M821T876_NEG

M836T846_POS

M855T580_POS

M590T142_POS

M806T804_2_PO

S

M771T653_POS

M971T1486_2_P

OS

M951T1264_POS

M750T604_POS

M797T790_POS

M1057T1538_PO

S

M969T1114_POS

M907T1441_POS

M718T682_POS

M943T1104_POS

M759T969_POS

M755T563_POS

M937T1295_POS

M903T1389_POS

M921T1200_POS

M917T1101_2_P

OS

M788T795_POS

M818T934_POS

M691T866_NEG

M809T682_POS

M905T652_POS

M969T1459_2_P

OS

M715T1248_POS

M1087T1576_PO

S

M885T1222_POS

M751T710_POS

M757T726_POS

M704T649_POS

M1238T738_POS

M733T770_POS

M1552T734_NE

G

M855T890_POS

M837T1422_POS

M703T765_POS

M849T580_NEG

M867T1115_POS

M908T737_NEG

M893T1488_POS

M860T907_NEG

M869T1188_POS

M745T793_NEG

M780T519_NEG

M648T490_POS

M779T611_POS

M1059T1558_PO

S

M817T674_POS

M819T726_POS

M845T1186_POS

M847T736_POS

M756T393_POS

M801T1023_POS

M791T640_NEG

M518T132_POS

M255T396_NEG

M893T835_POS

M875T882_1_PO

S

M854T681_NEG

M745T638_POS

M1085T1558_PO

S

M713T1078_POS

M830T930_POS

M833T1023_POS

M823T755_POS

M795T726_POS

M797T725_POS

M768T1023_POS

M689T807_NEG

M833T630_POS

M863T1303_POS

M805T584_POS

M665T973_NEG

M735T778_POS

M688T235_POS

M873T813_POS

M867T839_POS

M759T896_POS

M652T1383_POS

M731T829_POS

M786T781_POS

M815T781_POS

M744T1081_POS

M901T946_NEG

M796T1033_POS

M1011T1465_PO

S

M853T681_NEG
M875T595_NEG
M803T804_POS
M851T786_POS
M886T592_POS
M709T1036_NE
G
M957T1363_NE
G
M913T1288_POS
M702T614_POS
M949T1229_POS
M762T797_POS
M782T679_POS
M753T765_POS
M841T783_POS
M647T866_POS
M985T1468_POS
M874T944_NEG
M727T777_NEG
M720T626_POS
M1150T500_NE
G
M370T118_POS
M703T771_NEG
M645T866_NEG

M815T688_NEG

M718T561_POS

M1013T1471_PO

S

M820T829_NEG

M1137T879_POS

M919T1487_POS

M856T778_NEG

M750T672_NEG

M665T1035_POS

M806T723_POS

M934T635_POS

M847T815_POS

M765T659_POS

M743T749_POS

M764T574_NEG

M804T898_POS

M899T580_NEG

M474T136_NEG

M747T752_POS

M787T697_NEG

M767T747_POS

M637T824_POS

M746T1154_POS

M1236T737_NE

G

M949T1245_POS
M895T1175_POS
M867T739_NEG
M728T616_POS
M815T652_NEG
M885T1258_POS
M791T666_POS
M851T839_POS
M821T672_POS
M845T1092_NE
G
M739T1081_POS
M789T706_NEG
M881T930_POS
M829T914_POS
M830T695_POS
M749T710_NEG
M661T760_POS
M799T781_POS
M620T796_POS
M957T1407_POS
M865T767_POS
M975T1202_POS
M825T815_POS
M721T748_POS
M663T803_POS

M704T1100_POS

M947T1232_2_P

OS

M590T169_NEG

M651T996_POS

M888T675_NEG

M667T1350_POS

M810T727_POS

M845T858_NEG

M917T1054_POS

M828T721_NEG

M917T976_NEG

M927T1259_POS

M790T863_POS

M897T1262_POS

M851T813_POS

M819T755_POS

M791T711_POS

M959T1374_NE

G

M562T131_NEG

M775T853_POS

M818T806_NEG

M863T650_POS

M909T1473_POS

M746T744_POS

M739T634_POS

M716T624_POS

M755T841_NEG

M828T1179_POS

M440T437_POS

M859T1216_POS

M789T744_NEG

M799T705_POS

M895T1190_POS

M619T123_NEG

M814T873_POS

M828T881_POS

M933T1397_NE

G

M583T729_NEG

M829T878_POS

M849T762_POS

M753T828_POS

M701T765_NEG

M748T647_NEG

M849T747_POS

M890T675_NEG

M839T777_POS

M693T929_NEG

M1011T1441_PO

S

M903T1365_POS

M779T756_POS

M725T722_NEG

M905T1404_POS

M743T746_NEG

M769T696_POS

M912T652_NEG

M827T584_POS

M737T1018_POS

M767T735_NEG

M544T148_POS

M719T1344_POS

M877T645_NEG

M783T715_POS

M905T1352_NE

G

M912T634_NEG

M667T975_POS

M836T745_POS

M368T106_POS

M831T596_POS

M753T792_NEG

M811T1154_2_P

OS

M588T148_NEG

M769T626_POS

M951T1285_POS

M853T832_POS

M903T1335_POS

M805T733_NEG

M634T560_POS

M802T720_NEG

M539T730_POS

M817T699_NEG

M828T825_POS

M825T782_POS

M741T693_NEG

M864T902_POS

M829T811_POS

M923T1263_POS

M1031T1538_PO

S

M879T1404_POS

M808T779_POS

M546T169_POS

M789T637_NEG

M811T914_POS

M945T1177_POS

M925T1327_POS

M916T1457_POS

M863T737_POS

M510T465_POS

M665T858_POS

M840T841_POS

M789T663_NEG

M861T1278_POS

M835T744_POS

M815T1077_POS

M778T582_POS

M931T1323_NE

G

M478T161_POS

M770T1090_POS

M748T662_NEG

M895T1457_POS

M813T712_NEG

M813T696_NEG

M753T706_NEG

M806T651_POS

M797T689_POS

M931T1355_NE

G

M821T706_POS

M833T646_POS

M893T1102_POS

M843T780_NEG

M957T1349_NE

G

M765T748_NEG

M769T794_POS

M835T680_POS

M871T761_POS

M851T641_NEG

M781T828_POS

M772T1157_POS

M885T785_NEG

M777T788_POS

M735T711_POS

M867T1097_POS

M883T705_POS

M735T529_POS

M858T1156_POS

M854T1195_POS

M701T674_POS

M528T176_POS

M731T603_POS

M794T751_POS

M888T653_POS

M526T147_POS

M849T802_POS

M665T1266_POS

M835T702_POS

M849T842_POS

M909T711_POS

M759T758_POS

M903T593_POS

M655T930_POS

M478T189_NEG

M865T1457_POS

M807T769_NEG

M885T803_NEG

M775T745_POS

M741T1154_POS

M817T1130_POS

M834T863_NEG

M530T195_POS

M279T319_NEG

M735T981_POS

M809T732_POS

M566T149_POS

M687T802_POS

M879T605_POS

M480T189_POS

M476T161_NEG

M650T1458_POS

M520T1342_POS

M849T1193_NE

G

M722T615_POS

M524T147_NEG

M832T801_POS

M821T1268_POS

M512T552_POS

M837T721_POS

M751T765_NEG

M953T1336_POS

M773T853_NEG

M951T1326_POS

M528T195_NEG

M809T664_POS

M789T1051_POS

M803T791_POS

M526T176_NEG

M862T605_POS

M858T847_NEG

M818T828_NEG

M806T797_NEG

M908T593_POS

M883T1193_POS

M765T698_POS

M724T586_1_PO

S

M729T829_NEG

M649T930_POS

M1073T1567_PO

S

M985T911_NEG
M712T643_NEG
M847T752_POS
M775T603_NEG
M832T838_NEG
M899T1180_POS
M867T630_POS
M695T1386_POS
M795T699_POS
M921T1457_POS
M871T1239_POS
M861T704_POS
M977T1512_POS
M789T831_POS
M735T955_POS
M832T536_NEG
M679T906_NEG
M1017T1071_NE
G
M889T1340_POS
M718T476_POS
M833T1205_POS
M891T1019_POS
M711T974_NEG
M817T699_POS
M807T613_POS

M973T1232_POS

M859T781_NEG

M927T1317_POS

M797T755_POS

M807T641_POS

M1057T1475_PO

S

M824T897_POS

M867T1067_POS

M901T569_POS

M777T1085_POS

M777T590_POS

M693T1335_POS

M816T859_POS

M667T889_POS

M871T1122_POS

M841T803_POS

M846T873_NEG

M793T719_POS

M707T650_POS

M845T1239_2_P

OS

M1180T629_NE

G

M845T1103_POS

M889T1279_POS

M882T570_NEG
M959T1398_NE
G
M808T769_NEG
M669T967_POS
M843T1121_POS
M1015T1503_PO
S
M877T587_POS
M744T691_POS
M1045T1547_PO
S
M633T704_POS
M502T202_POS
M852T641_NEG
M931T1366_POS
M909T764_NEG
M795T745_POS
M768T1048_POS
M755T591_POS
M745T746_POS
M765T1090_POS
M796T1106_POS
M641T893_POS
M833T721_POS
M358T116_POS

M763T739_NEG
M959T1443_POS
M763T1048_POS
M796T1092_POS
M711T889_NEG
M979T1297_POS
M720T573_NEG
M803T669_NEG
M890T676_POS
M951T1299_POS
M838T859_POS
M871T1221_POS
M947T832_POS
M860T587_POS
M935T1472_POS
M843T801_NEG
M757T896_NEG
M739T676_NEG
M779T808_NEG
M769T684_NEG
M989T1002_NE
G
M807T684_POS
M829T704_NEG
M785T862_POS
M865T750_POS

M790T603_POS

M726T558_POS

M793T809_NEG

M955T1395_POS

M801T611_NEG

M747T794_POS

M793T1090_POS

M649T944_POS

M797T555_NEG

M1170T546_POS

M915T1281_POS

M836T833_POS

M819T744_NEG

M887T1287_POS

M502T155_POS

M949T1486_POS

M647T929_NEG

M803T1089_POS

M412T323_POS

M878T870_NEG

M804T774_NEG

M845T757_NEG

M1005T1344_PO

S

M893T1423_POS

M791T696_POS

M901T1316_POS

M862T934_NEG

M756T736_POS

M702T586_POS

M762T476_NEG

M554T147_POS

M717T733_NEG

M763T1023_POS

M813T626_NEG

M747T688_POS

M791T751_NEG

M858T834_NEG

M814T802_POS

M805T601_POS

M753T1120_POS

M344T110_POS

M781T1193_POS

M500T155_NEG

M283T502_NEG

M893T761_NEG

M731T833_NEG

M772T750_POS

M1009T1417_PO

S

M820T598_NEG

M835T665_POS

M858T587_NEG

M789T1031_POS

M743T601_POS

M791T1092_POS

M853T597_POS

M746T614_NEG

M848T808_POS

M784T722_POS

M797T777_POS

M848T898_NEG

M1588T650_NE

G

M845T1145_POS

M728T603_POS

M987T1475_POS

M805T1156_POS

M799T591_NEG

M830T748_POS

M882T785_NEG

M874T811_NEG

M879T702_NEG

M804T743_POS

M798T1165_POS

M882T1257_POS

M860T785_POS

M815T1043_POS

M867T806_POS

M985T1413_POS

M869T1125_POS

M832T880_POS

M825T797_POS

M979T1284_POS

M783T838_POS

M913T1220_POS

M763T661_NEG

M767T1156_POS

M791T1106_POS

M825T773_POS

M1554T744_NE

G

M981T1378_POS

M1340T856_POS

M833T1231_POS

M975T1488_POS

M849T1306_POS

M715T643_POS

M1168T546_NE

G

M801T757_POS

M839T699_NEG

M635T906_POS

M653T1039_POS

M831T1143_POS

M759T873_POS

M895T1157_POS

M720T540_POS

M695T1011_NE

G

M761T735_POS

M713T968_NEG

M919T1111_POS

M751T552_POS

M811T701_POS

M888T1422_POS

M899T1256_POS

M832T795_NEG

M726T538_POS

M635T776_POS

M867T1422_POS

M847T1220_POS

M719T701_POS

M508T202_POS

M882T587_POS

M917T1386_POS

M867T754_NEG

M798T749_POS

M803T577_POS

M869T785_NEG

M827T825_POS

M769T736_POS

M899T1292_POS

M807T825_POS

M827T642_NEG

M839T759_POS

M609T736_POS

M860T613_NEG

M872T881_NEG

M858T1311_POS

M877T1359_POS

M753T774_NEG

M893T1076_POS

M884T592_NEG

M929T1310_NE

G

M881T719_NEG

M763T1065_POS

M829T684_NEG

M872T757_NEG

M861T759_POS

M847T1260_POS

M828T868_POS

M813T933_POS

M693T944_NEG

M851T914_POS

M743T693_POS

M789T814_1_PO

S

M810T639_NEG

M677T1004_POS

M791T718_NEG

M987T1442_POS

M574T167_POS

M665T869_NEG

M773T553_NEG

M667T917_POS

M790T743_NEG

M830T780_NEG

M622T849_POS

M833T665_POS

M771T761_POS

M780T710_NEG

M807T1223_POS

M859T1027_POS

M861T1294_POS

M695T956_NEG

M985T1442_POS

M955T1376_POS

M806T733_NEG

M659T732_POS

M827T658_NEG

M779T710_NEG

M789T775_POS

M819T1102_POS

M886T1366_POS

M799T897_POS

M921T1076_POS

M943T1343_POS

M741T740_POS

M480T202_POS

M821T1240_POS

M1017T1525_PO

S

M691T676_POS

M847T1243_POS

M751T1053_POS

M862T661_NEG

M719T710_NEG

M979T1337_POS

M921T1169_POS

M1001T1290_PO

S

M651T1011_POS

M867T1134_POS

M767T659_NEG

M734T736_POS

M895T1133_POS

M876T801_NEG

M804T669_NEG

M721T788_POS

M791T687_NEG

M818T698_NEG

M933T1439_POS

M651T955_POS

M923T1487_POS

M828T658_NEG

M812T779_POS

M665T1051_POS

M793T1166_POS

M816T751_NEG

M785T684_POS

M783T725_POS

M832T560_NEG

M699T930_NEG

M804T593_POS

M795T1232_POS

M895T813_NEG

M765T699_NEG

M905T1240_POS

M799T765_POS

M864T704_NEG

M801T625_NEG

M782T688_NEG

M919T1070_POS

M872T866_NEG

M858T551_NEG

M789T1069_POS

M878T1133_POS

M637T796_POS

M717T692_POS

M1071T1548_PO

S

M793T691_POS

M774T656_NEG

M732T713_POS

M823T740_POS

M777T656_NEG

M516T146_POS

M811T871_POS

M1005T1536_PO

S

M783T1267_POS

M676T512_POS

M875T1340_POS

M869T772_NEG

M751T746_NEG

M781T577_POS

M781T600_POS

M316T96_POS

M921T1148_POS

M773T800_POS

M855T718_NEG

M876T881_NEG

M929T1369_POS

M811T719_POS

M860T860_NEG

M771T800_NEG

M764T625_NEG

M886T902_NEG

M665T796_POS

M757T653_POS

M828T758_POS

M989T1501_POS

M727T588_POS

M841T755_NEG

M836T597_POS

M1043T1526_PO

S

M623T934_POS

M845T1220_POS

M783T643_POS

M757T612_POS

M817T1103_POS

M717T688_POS

M945T1378_NE

G

M838T785_POS

M851T613_NEG

M880T1195_POS

M1073T1501_PO

S

M865T1047_POS

M811T1331_POS

M803T599_POS

M759T693_POS

M635T747_POS

M767T681_POS

M767T687_POS

M685T893_NEG

M793T1194_POS

M657T949_POS

M819T1185_POS

M711T1015_POS

M839T743_POS

M945T1109_POS

M609T899_POS

M639T861_NEG

M884T840_NEG

M854T727_NEG

M826T808_POS

M991T1525_POS

M836T646_NEG

M837T1300_POS

M664T541_POS

M875T1311_POS

M803T694_NEG

M729T552_POS

M773T774_NEG

M927T1249_NE

G

M685T1012_POS

M805T663_POS

M797T743_NEG

M834T779_POS

M899T1312_POS

M869T797_NEG

M843T1043_POS

M873T1168_NE

G

M805T715_NEG

M983T1376_POS

M850T868_POS

M779T1152_POS

M755T665_POS

M757T626_POS

M805T824_NEG

M833T774_NEG

M797T555_POS
M953T1284_POS
M716T1015_POS
M703T528_POS
M835T1276_POS
M1059T1501_PO
S
M787T730_POS
M649T1011_NE
G
M845T1130_POS
M625T961_POS
M897T1231_POS
M857T1168_POS
M825T576_NEG
M819T1168_POS
M759T779_POS
M813T762_POS
M733T663_POS
M833T691_POS
M858T802_NEG
M891T781_POS
M861T1243_POS
M716T1043_POS
M740T681_POS
M779T827_NEG

M869T1155_POS

M661T779_POS

M778T734_NEG

M767T1177_POS

M823T1320_POS

M769T712_POS

M915T1340_POS

M502T177_NEG

M769T759_NEG

M951T1231_POS

M773T699_POS

M839T1381_POS

M869T816_NEG

M884T1319_POS

M927T1351_POS

M832T729_NEG

M929T1397_POS

M827T625_NEG

M781T667_POS

M965T1525_POS

M830T1243_POS

M683T952_POS

M807T1203_POS

M859T654_POS

M715T687_NEG

M887T1385_POS

M314T91_POS

M1003T1514_PO

S

M804T696_NEG

M857T743_NEG

M719T740_POS

M831T1180_POS

M746T585_NEG

M667T934_NEG

M844T812_NEG

M941T1072_POS

M925T1314_POS

M777T765_NEG

M645T412_2_PO

S

M709T952_POS

M681T973_NEG

M813T740_POS

M780T723_POS

M783T660_POS

M834T597_NEG

M538T145_NEG

M811T686_NEG

M785T705_POS

M827T772_1_PO

S

M881T662_POS
M860T1359_POS
M864T662_POS
M903T1301_NE
G
M955T1350_POS
M937T1501_POS
M870T808_NEG
M859T740_POS
M808T583_NEG
M681T891_POS
M817T1144_POS
M927T1293_POS
M825T1358_POS
M871T1196_POS
M883T776_NEG
M827T601_POS
M923T1231_POS
M841T737_NEG
M781T839_NEG
M506T161_POS
M863T1333_POS
M712T997_NEG
M843T1156_POS
M772T603_NEG
M797T1287_POS

M795T777_NEG

M800T839_POS

M639T849_POS

M853T861_POS

M855T646_POS

M839T744_NEG

M743T669_NEG

M639T1000_POS

M841T689_NEG

M456T361_POS

M841T1066_POS

M759T734_POS

M884T605_1_PO

S

M733T763_POS

M897T1194_POS

M949T1168_POS

M871T1180_POS

M925T1292_POS

M814T834_POS

M971T1384_NE

G

M851T1359_POS

M847T1168_POS

M865T1381_POS

M901T1243_NE

G

M741T735_POS

M819T1220_POS

M873T1291_POS

M822T812_POS

M920T1027_1_P

OS

M855T911_POS

M883T759_NEG

M901T1349_POS

M1266T752_NE

G

M919T1376_NE

G

M776T712_NEG

M340T109_POS

M829T613_POS

M783T804_POS

M641T917_POS

M891T1381_POS

M653T898_NEG

M761T716_POS

M765T680_NEG

M855T690_NEG

M856T1252_POS

M947T1111_POS

M825T748_POS

M791T1143_POS

M783T625_POS

M837T689_NEG

M709T1052_NE

G

M758T722_POS

M719T624_POS

M779T625_POS

M478T203_NEG

M865T706_NEG

M811T858_POS

M797T737_POS

M831T729_NEG

M842T749_NEG

M837T741_POS

M809T1290_POS

M759T670_POS

M927T1335_POS

M825T599_NEG

M971T776_POS

M800T812_POS

M895T804_NEG

M554T664_NEG

M819T862_NEG

M961T1474_POS

M883T740_NEG

M494T145_POS

M830T812_POS

M745T716_NEG

M963T1500_POS

M550T189_POS

M746T528_NEG

M771T729_NEG

M621T869_POS

M809T1268_POS

M705T587_POS

M797T1301_POS

M835T1293_POS

M873T1252_POS

M707T895_POS

M1011T1413_PO

S

M737T1046_POS

M637T973_POS

M919T1422_POS

M853T1404_POS

M1498T686_NE

G

M281T410_NEG

M595T862_POS

M741T677_POS

M963T1474_POS

M816T647_NEG

M859T1233_POS

M917T1331_NE

G

M760T888_POS

M923T1133_POS

M757T710_POS

M823T1302_POS

M730T657_POS

M853T663_NEG

M837T1332_POS

M635T973_NEG

M655T880_POS

M931T1407_POS

M808T685_POS

M722T578_NEG
